# Allelic variants of a potato *HEAT SHOCK COGNATE 70* gene confer improved tuber yield under a wide range of environmental conditions

**DOI:** 10.1002/fes3.377

**Published:** 2022-03-15

**Authors:** Raymond Campbell, Laurence Ducreux, Graham Cowan, Vanessa Young, Gift Chinoko, Gloria Chitedze, Stanley Kwendani, Margaret Chiipanthenga, Craita E. Bita, Obed Mwenye, Hassan Were, Lesley Torrance, Sanjeev Kumar Sharma, Robert D. Hancock, Glenn J. Bryan, Mark Taylor

**Affiliations:** ^1^ Cell and Molecular Sciences The James Hutton Institute Dundee UK; ^2^ James Hutton Limited Dundee UK; ^3^ International Potato Centre Lilongwe Malawi; ^4^ Department of Agricultural Research Services Bvumbwe Agricultural Research Station Limbe Malawi; ^5^ Department of Agriculture and Land Use Management Masinde Muliro University of Science and Technology Kakamega Kenya; ^6^ School of Biology Biomolecular Sciences Building University of St Andrews St Andrews UK

**Keywords:** abiotic stress, field trial, HEAT SHOCK COGNATE 70, potato, promoter, yield

## Abstract

Previously, we developed and applied a glasshouse screen for potato tuber yield under heat stress and identified a candidate gene (*HSc70*) for heat tolerance by genetic analysis of a diploid potato population. Specific allelic variants were expressed at high levels on exposure to moderately elevated temperature due to variations in gene promoter sequence.

In this study, we aimed to confirm the results from the glasshouse screen in field trials conducted over several seasons and locations including those in Kenya, Malawi and the UK. We extend our understanding of the *HSc70* gene and demonstrate that expression level of *HSc70* correlates with tolerance to heat stress in a wide range of wild potato relatives. The physiological basis of the protective effect of HSc70 was explored and we show that genotypes carrying the highly expressed *HSc70* A2 allele are protected against photooxidative damage to PSII induced by abiotic stresses. Overall, we show the potential of *HSc70* alleles for breeding resilient potato genotypes for multiple environments.

## Graphical abstract

Potato is a critical crop for food security; however, it is vulnerable to reduced tuber yield at even moderately elevated temperature. In this work, we show the potential of HSc70 alleles for breeding resilient potato genotypes for multiple environments.

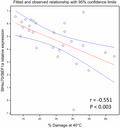

## 1 INTRODUCTION

Potato is the third most important food crop in the world after rice and wheat and has a central role in global food security (Birch et al., 2012). In general, potato is a cool climate crop, particularly sensitive to heat and drought stresses (Dahal et al., 2019; George et al., 2017)., There is, however, a significant variation in response to heat stress between cultivars (Levy, 1986; Levy et al., 1991), in land races and wild potato species (Hetherington et al., 1983; Reynolds & Ewing, 1989), and in progeny clones from heat‐tolerant parents (Haynes & Haynes, 1983; Veilleux et al., 1997). Heat and drought conditions can cause yield losses of up to 70% and induce tuber sprouting and secondary growth that cause further losses due to decline in tuber quality (FAOSTAT, 2017). Factors that impact on potato yield in different environments are not well understood, however, maintaining yield under stress conditions is one of the highest priorities of modern agriculture.

Whilst there is considerable variation in the response of potato genotypes to stress conditions, genetic dissection of abiotic stress responses remains a challenge in potato, as well as in most other crop species. With the advent of improved genetic and genomic tools, however, some progress has been made recently using diploid potatoes to simplify genetic analysis (Marand et al., 2019; Prashar et al., 2014). Particularly useful in potato genetic studies has been a highly heterozygous biparental diploid population (06H1) that segregates for many traits important to breeding activities including tuber shape and eye depth (Prashar et al., 2014), virus resistance (Torrance et al., 2020) and heat tolerance (Trapero‐Mozos, Ducreux, et al., 2018). Importantly, this population does not segregate for functional variation in *StCDF‐1*, the gene underlying a large effect QTL for foliage maturity on chromosome 5 (Kloosterman et al., 2013) and so any effects on heat tolerance are not related to changes in foliage maturity. In the 06H1 population, we identified a large effect QTL on chromosome 4 that impacted on tuber yield at both normal and elevated temperatures deploying a model tuberization system (Trapero‐Mozos, Ducreux, et al., 2018). A candidate gene encoding HEAT SHOCK COGNATE 70 (HSc70) was identified within one of the three QTL intervals associated with elevated yield. *HSc70* allelic variants were expressed at different levels, and the level of expression was positively linked to elevated tuber yield in the 06H1 progeny and in transgenic potato lines that over‐expressed *HSc70*. Transient expression of potato *HSc70* alleles in *Nicotiana benthamiana* resulted in significantly enhanced tolerance to elevated temperature, as assessed by a membrane damage assay based on electrolyte leakage in leaves. These results identified *HSc70* expression level as a significant factor influencing yield stability under moderately elevated temperature and identified specific allelic variants of *HSc70* for the induction of thermotolerance via conventional introgression or molecular breeding approaches.

As the *HSc70* gene was identified in a model tuberization system, an important aim of this study was to investigate the tuber yield performance of genotypes carrying different *HSc70* allelic variants in potato field trials conducted in different environments. Here, we report the results of field trials from sites in Kenya, Malawi and the UK, where the African sites were significantly warmer and drier than the UK sites. In both conventional QTL analysis of the 06H1 population and genome‐wide association studies (GWAS) of a panel containing 290 tetraploid varieties, we tested the hypothesis that there is a tuber yield QTL in the region where *HSc70* maps when these populations were trialled under UK conditions. This would indicate that variation in the *HSc70* gene is a significant factor in potato tuber yield, not only in warm dry environments but also in temperate zones. In addition, we aim to extend our observations from the diploid 06H1 population to a wide range of potato wild relative species in order to determine whether there is any correlation between *HSc70* expression level and leaf membrane damage of exposure to elevated temperature. We hypothesise that the alleles that are expressed at higher levels are associated with lower damage from elevated temperature. Taken together, the work presented here points to the potential for deploying markers associated with different *HSc70* alleles for breeding genotypes with improved tuber yield in a wide range of environmental conditions.

## 2 MATERIALS AND METHODS

### 2.1 Field trialling, field preparations and agronomic practices

A subset of 60 clones from the 06H1 population were field trialled at Sang'alo, Kenya (0° 31′ N 34° 36′ 0′′ E), and Makoka, Malawi (35 11′ 0′ E, 15 32′ 0′ S), between 2018 and 2020. The weather conditions at the two sites are presented in Table 1: Makoka is generally warmer and drier than the Sang'alo site. Glasshouse produced, disease and virus‐free mini tuber seed was grown at The James Hutton, Invergowrie, UK, during both seasons. Trials were organised as 3 plant plots per genotype, replicated 4 times within a random block design. Tubers were planted 30 cm apart in pre‐made drills with 1‐m distance between plots during the 2nd week of March at Sang'alo and the 4th week of December at the Makoka trials. All field trials went through standard agronomic practices for fertiliser and pesticide application.

**TABLE 1:** Climatic conditions during the trials in Kenya (Sang'alo) and Malawi (Makoka)

Month	Max	Min	Rainfall	Wet days	Month	Max	Min	Rainfall	Wet days
*Makoka 2018/2019*					*Makoka 2019/2020*				
Dec	27.8	18.9	263.3	19	Dec	28.7	19.3	307.3	14
Jan	27.4	19.1	228.1	22	Jan	26.7	18.8	339.1	21
Feb	27.8	18.5	204.4	13	Feb	27.6	18.9	131.6	17
Mar	26.9	17.8	299.3	13	Mar	27.9	17.1	28	3
Apr	26.6	16.5	32.1	7	Apr	26.9	15.5	2.3	1
*Sang'alo 2018*					*Sang'alo 2019*				
Mar	27	16	372.3	31	Mar	32	17	90.3	18
Apr	25	15	455	30	Apr	29	16	251.7	28
May	25	15	451.9	31	May	27	15	381	31
Jun	24	14	286.4	30	Jun	24	15	189.1	30
Jul	24	14	191.5	30	Jul	24	15	117.5	30

### 2.2 Field trials in the UK

The mapping population described (06H1) is a full‐sibling progeny [n =186 of a cross between two highly heterozygous diploid potato clones (HB171(13) and 99FT1b5)], both of which result from crosses between diploids of *Solanum tuberosum* group Tuberosum and *Solanum tuberosum* group Phureja. Field trials were carried out as five plant plots per genotype replicated twice using alpha designs between 2009 and 2011 at Balruderry Farm, near Invergowrie, Dundee (56° 28′ N, 03° 03′ W, 132 M above sea level). Trialling of 186 clones and the two parents was performed based on a two replicate randomised block design and seed tubers were planted in the last week of April, 30 cm apart in pre‐made drills with 2‐m distance between plots. All field trials went through standard agronomic practices for fertiliser and pesticide application.

### 2.3 QTL analysis

The 06H1 parental and progeny clones were previously genotyped with an Illumina Infinium 8k Potato SNP Array (Felcher et al., 2012; Hamilton et al., 2011). Yield data from trials performed in years 2009–2011 were used for QTL analysis with the linkage map reported by Prashar et al., 2014. QTL mapping was performed deploying MapQTL® 6.0 (Van Ooijen, 2009) and Genstat 19th Edition (VSN International, 2019). The non‐parametric Kruskal–Wallis (KW) test supported in MapQTL version 6.0 was performed initially. In the KW method, a single‐marker analysis is deployed to test the association of a marker with the trait at significance *p* ≤ 0.001 (Kruskal & Wallis, 1952). The identified QTL regions were further explored with single‐trait‐single‐environment QTL analysis in Genstat. This was done by simple interval mapping (SIM) followed by Composite Interval Mapping (CIM), controlling the effects of chromosomes onto the QTL being tested and so increasing the precision of QTL detection Zeng (1994). The Genstat procedure for analysing trial data from multiple environments QMQTLSCAN, QCANDIDATES and QMBACKSEL were used for candidate QTL identification and selection, and QMESTIMATE was deployed for QTL model fitting. A ‘3 environment’ model was adopted using the three trial years as the three environments.

### 2.4 Genome‐Wide Association Study (GWAS)

GWAS was conducted with the dataset reported by Sharma et al. (2018), in order to determine tuber yield QTL in a panel of tetraploid potato varieties, trialled in the UK. In brief, the association panel comprised 351 diverse tetraploid genotypes and of these 290 clones, for which complete field trial data were available, were included in the current study. Yield data obtained from replicated multi‐environment trials involving two locations (Cambridge and York, United Kingdom) trialled over two consecutive years (2012 and 2013) were used for performing GWAS. Best Linear Unbiased Estimates (BLUEs) for yield were calculated by modelling genotype as a fixed effect and treating all other effects as random by applying REML implemented in Genstat (VSN International Limited, http://www.vsni.co.uk). Genotyping, employing genomic DNA extracted from young leaf tissue from individual field grown plants, was performed with the Infinium 8k Potato SNP Array (Felcher et al., 2012; Hamilton et al., 2011) according to the manufacturer's protocols. SNP allelic dosages (genotypes) were called using fitPoly R package which resolved genotypic classes for 6401 SNPs. Of these, 5885 robust SNPs were retained for GWAS after filtering for monomorphic SNPs and those displaying higher (>20%) missing data and lower (<5%) minor allele frequencies (MAF). SNP genomic annotation was revised according to the recently released potato genome assembly v6.1 (Pham et al., 2020).

GWAS was performed with the GWASpoly R package (Rosyara et al., 2016) employing all genetic effect models plausible in tetraploids, namely (1) additive, (2) simplex dominance, (3) duplex dominance, (4) diplo‐general, (5) diplo‐additive and (6) general as described in GWASpoly manual. All genetic models were further evaluated using four different statistical models viz. (i) Naïve model, without controlling any confounding effects, (ii) Kinship model, controlling just for individual relatedness (K), (iii) Population Structure model, controlling for population structure (Q) effects only and (iv) Full model, accounting for K as well as Q confounding effects. All these four models are hereafter referred to as Naïve, K, Q and QK models respectively. Within each genetic model, fitness of different statistical models was evaluated by Quantile–Quantile (Q–Q) plots of the observed versus expected –log10(*p*) values which should follow a uniform distribution under the null hypothesis. Models were ranked according to the genomic control inflation factor (λ_GC_) metric calculated as the median of the resulting chi‐squared test statistics divided by the expected median of the chi‐squared distribution. The false discovery rate (FDR) correction (with genome‐wide α = 0.10) method in GWASpoly was applied for establishing a p‐value detection threshold for statistical significance. For all GWAS models, K and QK models outperformed Naïve and Q models (Figure S4a,b). The efficacy of K and QK models was largely indistinguishable, and all further results and discussion are inferred from these two GWAS models only.

### 2.5 Growth of potato wild species

26 accessions representative of wild diploid *Solanum* species were obtained from the Commonwealth Potato Collection (Bradshaw & Ramsay, 2005). Plants were propagated from stem cuttings in 90‐mm Petri dishes containing MS medium (Murashige & Skoog, 1962) supplemented with 20 g/L sucrose and 8 g/L agar at 18 ± 4°C, 16 h light, 8 h dark, and light intensity 100 μmol m^−2^ s^−1^. Four weeks after subculture, in vitro plantlets were transferred to 9‐cm diameter pots containing a standard compost mix. Plants were grown in a glasshouse maintained at a daytime temperature of 18f°C and a nocturnal temperature of 16°C for 2 weeks. Light intensity (photosynthetic photon flux density) ranged from 400 to 1000 μmol m^−2^ s^−1^. Then, the plants were acclimated for 1 week under controlled environment conditions (20 C day/16°C night −12 h light). A sub‐sample of the plants was moved to a cabinet at high temperature (40°C – 12 h light) or maintained at 20°C day/16°C night at a light intensity of 150 μmol m^−2^ s^−1^. After 24 h, leaves were assayed for membrane damage or frozen in liquid nitrogen and then stored at −80°C until required.

### 2.6 Electrolyte leakage assay

Membrane damage was assessed with an electrolyte leakage assay (Campos et al., 2003). Six replicate samples of four 10‐mm leaf discs from the 4th node were punched from a single leaf per sample assayed and placed in a 50‐ml tube. The discs were washed twice with de‐ionised water, blotted dry and returned to the tube with 5 ml of de‐ionised water. Following gentle shaking for 1 h at 29°C, 25 ml de‐ionised water was added, and the initial conductivity was measured using a conductivity meter (Model HI99300, Hanna Instruments Ltd, Leighton Buzzard, UK). Samples were autoclaved, and total conductivity was determined after cooling to room temperature. The percentage of injury (membrane damage) was calculated as initial conductivity x 100 / total conductivity.

### 2.7 Chlorophyll fluorescence measurements

Chlorophyll fluorescence measurements were made on leaves from 06H1 genotypes grown under control and stress conditions to determine whether differences in photosystem II (PSII)_ performance correlated with *HSc70* genotype. Measurements of maximum quantum yield of PSII (Fv/Fm) were conducted with a Handy PEA fluorimeter (Hansatech Instruments Ltd., Norfolk, UK). Terminal leaflets on the 4th node were dark adapted for 20 min prior to the collection of fluorescence parameters following a saturating light pulse of 3000 µmol m^−2^ s^−1^.

### 2.8 qRT‐PCR

RNA was extracted from potato leaves using a Qiagen RNAeasy extraction kit. The first‐strand cDNA templates were generated by reverse transcription, deploying RNA to cDNA EcoDry Premix (Takara Bio Group). Potato elongation factor1‐alpha (EF1α) primers were used as a control. The expression level of *HSc70* was analysed with the StepOnePlus Real‐Time PCR system (Applied Biosystems) and StepOne Software version 2.3 (Applied Biosystems, Foster City, CA, USA). Gene‐specific primers and Universal probe Library (UPL, Roche Life Science) probes were used at a concentration of 0.2 and 0.1 μM respectively. Thermal cycling conditions were as follows: 10‐min denaturation at 95°C followed by 40 cycles of 15 s at 94°C, 60 s at 60°C. Relative expression levels were calculated, and the primers validated using the Ct method (Livak & Schmittgen, 2001). To normalise the values, an alternative method for calculating relative quantification was applied (Pfaffl, 2001).

### 2.9 Cloning and in silico promoter analysis of *Hsc70* promoter sequence in potato wild species

Primers were designed to amplify promoter fragments of the *HSc70* gene from12 wild species accessions, starting approximately 1000 bp upstream of the translational start codon. The aim was to determine whether any promoter sequences were associated with the expression level of the *HSc70* gene. PCR of genomic DNA was conducted with Platinum™ Taq DNA Polymerase (ThermoFisher) under the following PCR cycling conditions: 94°C for 30 s, 94°C for 15 s, 55°C for 30 s and 68°C for 1 min and 30 cycles. This fragment was cloned into pGEM‐Teasy (Promega). Plasmid DNA was isolated using the Promega Wizard Plus minipreps DNA Purification system. DNA from a minimum of 12 independent colonies was sequenced with M13 forward and reverse primers. In silico promoter analysis was carried out on the obtained sequences deploying the PLACE database (http://www.dna.affrc.go.jp/PLACE/signalscan.html; Higo et al., 1999).

## 3 RESULTS

### 3.1 Tuber yield in field trials in Kenya and Malawi

A total of 60 genotypes from the 06H1 population were chosen for field trials in Kenya (Sang'alo, Bungoma region, 0° 31′ N 34° 36′ 0′′ E, 1920 M above sea level) and Malawi (Makoka, Zomba region, 35 11′ 0′ E,15 32′ 0′ S, 1029 M above sea level). All genotypes were selected for PVY resistance based on both resistance data and the presence of a SNP marker (c2_22749) tightly linked to a resistance gene on LG 9 described in Torrance et al. (2020). The selection was also based on the allelic composition of the *HSc70* gene (Soltu.DM.04G007430.1), previously identified as a candidate gene underpinning a QTL for tuber yield in a model tuberization system grown at 28°C (Trapero‐Mozos, Morris, et al., 2018). Four alleles designated as A1, A2, A3 and A4 were identified in the parents (A1/A2 in female parent HB171(13) and A3/A4 in male parent 99FT1b5) and genotypes from each of the 4 possible genotype classes (12 A1/A3, 14 A1/A4, 15 A2/A3 and 19 A2/A4) were selected.

**FIGURE 1:**
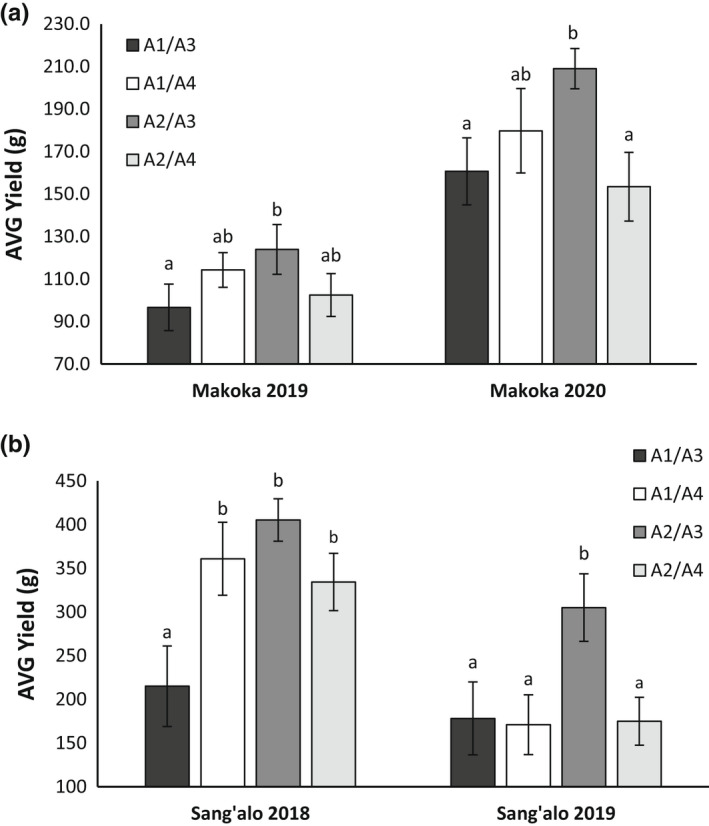
Yield genotypic means of 06H1 genotypes grown during 2 seasons in Malawi (Makoka, a) and Kenya (Sang'alo, b). Genotypes are grouped according to their *Hsc70* allelic composition. The data are represented as average FW yield (g) ± standard error of the mean (*n* = 6) from randomised trials harvested at 70 days post emergence. Letters in the bar chart (a, ab, b) represent significant differences (one‐way ANOVA and Fisher's Unprotected LSD test: *p* < 0.05)

Tuber yield was assessed from randomised, replicated trials in harvests at 70 and 90 days after planting and there was no significant additional yield increase after 70 days (Figure S1), and so the data shown is for the 70‐day harvest (Figure 1a,b).

Overall, there was considerable variation in tuber yield both between the two growth seasons and between growth sites, with the 2019 trials showing much lower yields than the 2020 trial (Makoka) and 2018 trial (Sangalo). The highest yields were measured in the 2018 Sangalo trial, with much lower overall yields in all other trials. The A2/A3 genotypes gave a significantly higher tuber yield in all four trials whereas the A1/A3 generally gave lower yields (significant in the 2019 Makoka trial and 2018 Sang'alo trial).

For the Makoka trials (Figure S2a), the tuber yields for each genotype was significantly correlated for the 2019 and 2020 trials, indicating consistent performance of the genotypes. In contrast, at the Sang'alo site (Figure S2b), there was only a weak correlation in yield performance between the seasons. The weather conditions during the trials are presented in Table 1 and indicate the different temperature conditions between the two Sang'alo trials, providing a possible explanation for the variation in performance. The early season conditions in 2019 were atypically hot and dry making this trial particularly challenging.

Overall, the results of these field trials confirm the results from a model tuberization system indicating associations between alleles of the HSc70 gene and tuber yield over two seasons and locations.

### 3.2 Tuber yield in field trials in the UK

**FIGURE 2:**
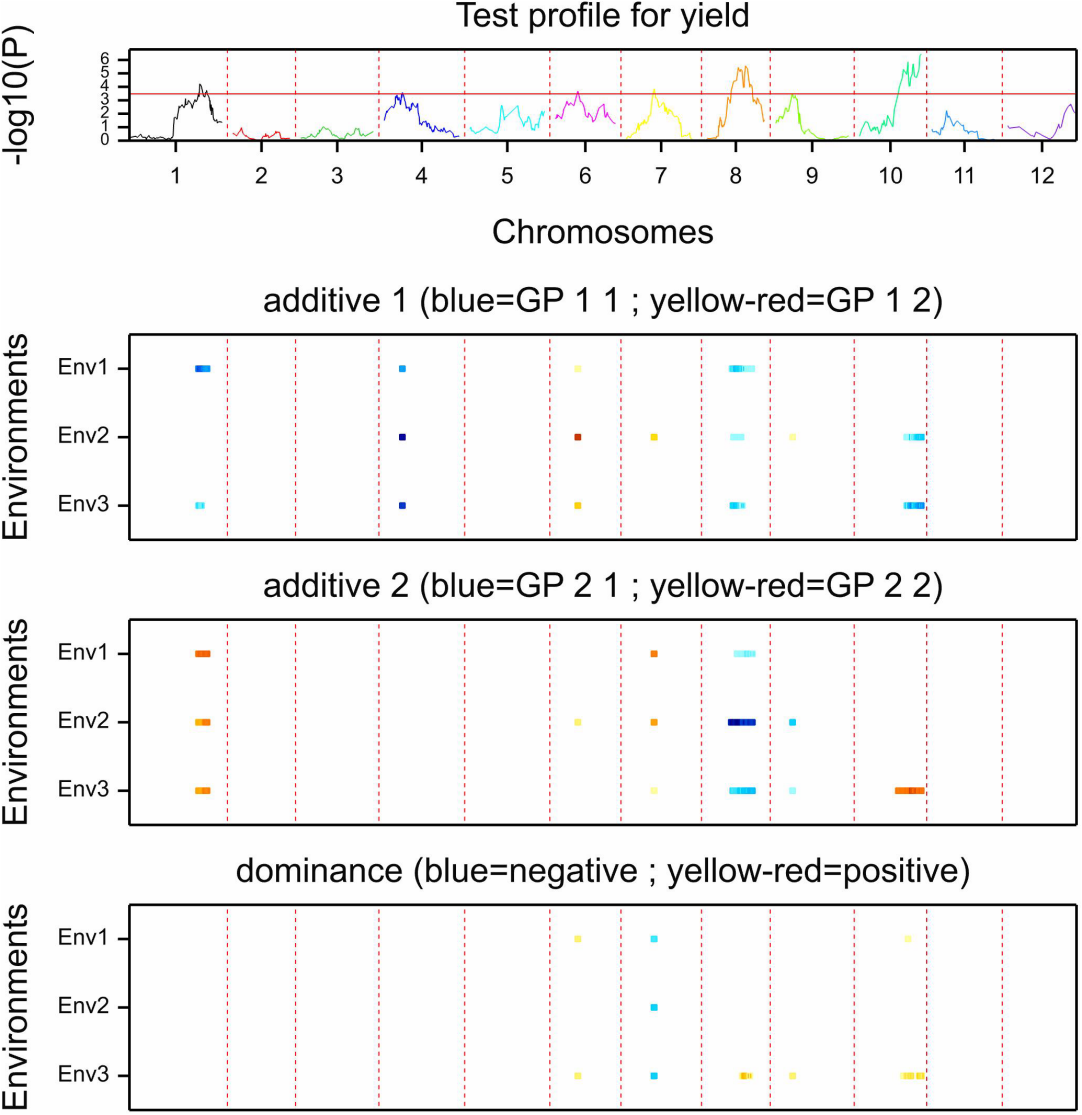
QTL plot for yield across 3 seasons (2009–2011) in 06H1 population. Top panel shows QTL profile across the 12 linkage groups. Panels show magnitude of significant additive (panel 2 for maternal parent, panel 3 for paternal parent) and dominance (panel 4) effects over the three growth seasons (Env 1 = 2009, Env2 = 2010, Env = 2011). A consistent additive maternal effect can be seen in all three seasons on chromosome 4

The 06H1 population comprising 186 clones was grown in the UK (Balruddery field site, Angus, Scotland 56° 28′ N, 03° 03′ W, 132 M above sea level), and phenotypic data were collected for several traits from 2009 to 2011 (V Young, MSc thesis). The mean tuber yield data in the three years showed correlations of 0.65 to 0.68 for the entire population and from 0.73 to 0.76 if only the 60 clones selected for growth in Africa were included (Figure S3). QTL analysis revealed many genetic effects for a wide range of traits (V. Young, MSc thesis) including final tuber yield. QTL analysis of yield data for 2009, 2010 and 2011 using the non‐parametric Kruskal–Wallis (KW) test revealed QTL effects for tuber yield on several linkage groups (data not shown). This dataset enabled us to investigate whether any tuber yield QTL were present in the region where *HSc70* maps. The marker most closely associated with yield in these analyses was solcap_snp_c2_54710 which mapped to 16.2 cM on LG 4 in the 06H1 linkage map. Simple and Composite interval mapping were subsequently performed by Genstat QTL analysis with a ‘3 environment’ model for the years 2009, 2010 and 2011, and detected a significant QTL effect at the location of SNP marker solcap_snp_c1_11135, which maps to 16.7 cM on the 06H1 linkage map and ~11Mb on the genome assembly, very close to the location of the marker detected by the non‐parametric method and close to the location of the *HSc70* gene (Figure 2). This LG 4 marker exhibits a significant maternal effect on yield (average +0.63 ± 0.13 kg) in all three years, compatible with the effect being due to segregation of the A2 HSc70 allele in the progeny.

**FIGURE 3:**
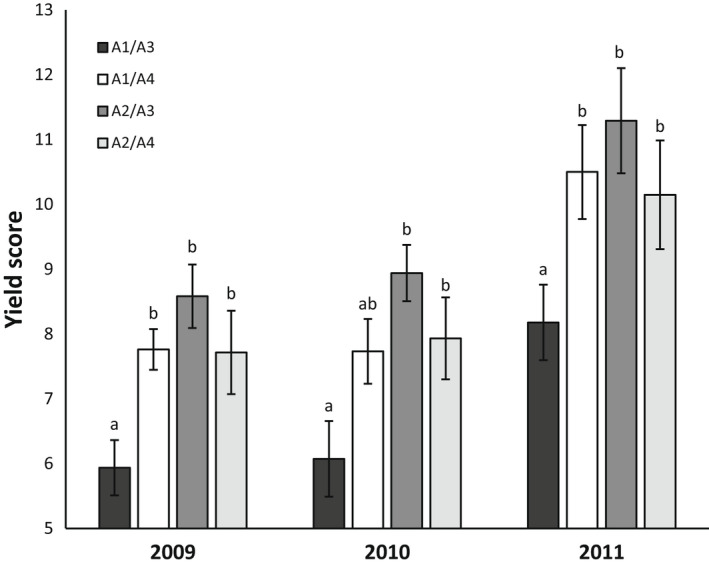
Yield genotypic means of 06H1 genotypes grown during 3 seasons at Balruddery field site, Angus, Scotland. Genotypes are grouped according to their *Hsc70* allelic composition. The data shown are the total FW yield (kg) ± standard error of the mean (*n* = 10) from randomised trials harvested between 139 and 151 days post planting. Letters in the bar chart (a, ab, b) represent significant differences (one‐way ANOVA and Fisher's Unprotected LSD test: *p* < 0.05)

The tuber yield scores for the 06H1 *HSc70* genotypic classes were calculated from these field trial data. As in the trials conducted in Kenya and Malawi, the A2/A3 group consistently gave a significantly higher tuber yield whereas the A1/A3 class gave the lowest yield (Figure 3). As with the field trials under warm, dry conditions in Kenya and Malawi, analysis of the 06H1 population under field conditions in the UK also identified a tuber yield QTL on chromosome 4 where *HSc70* maps. This supports the hypothesis that the locus in the 06H1 population is important for tuber yield under different environments.

### 3.3 UK GWAS panel field trials

**FIGURE 4:**
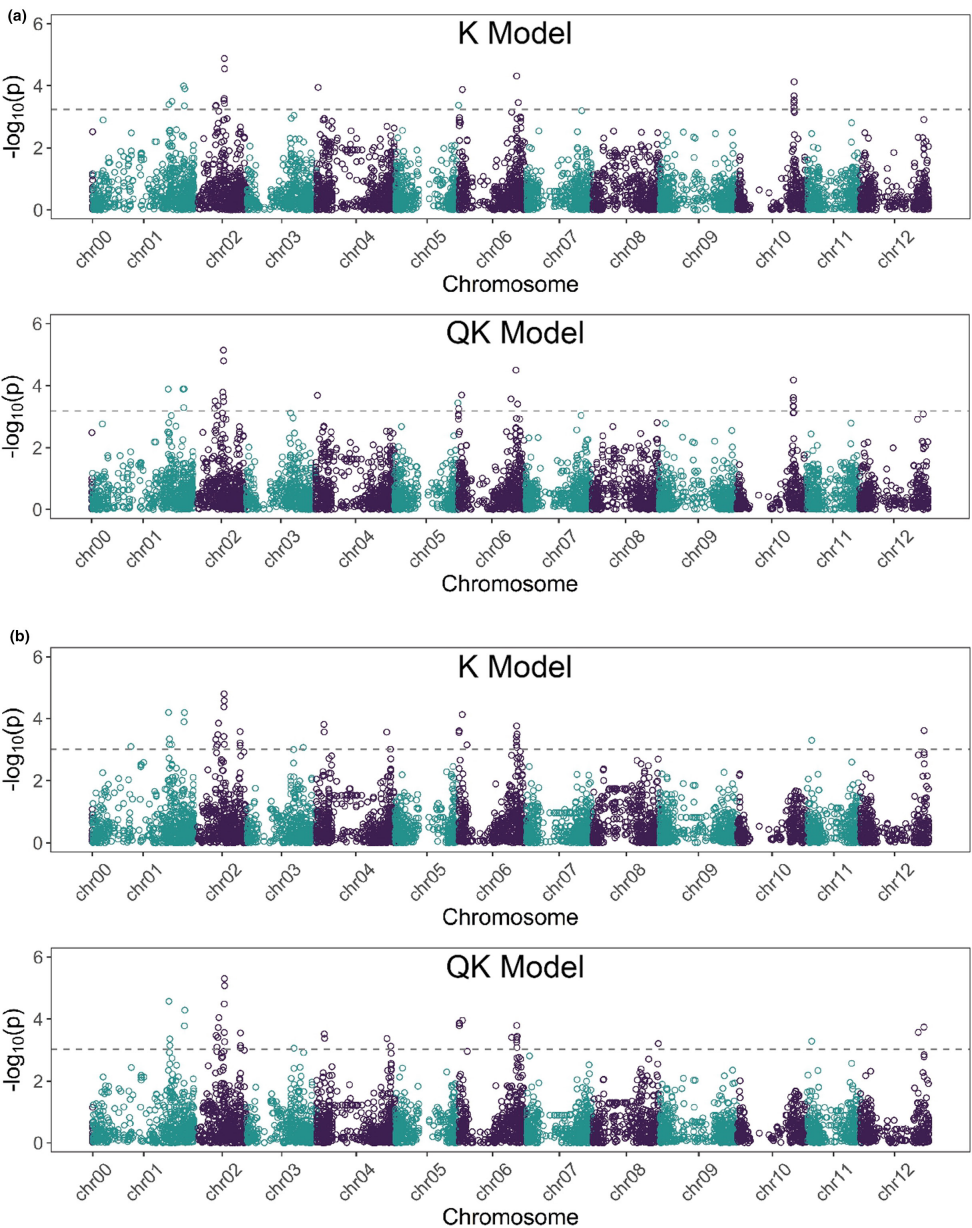
GWAS Manhattan plots for ‘general’ (A) and ‘diplo‐general’ (B) genetic effect models with significant tuber yield QTL on chromosome 4. Significance threshold (dashed line) is the genome‐wide false discovery rate (FDR) correction (α = 0.10) method

Tuber yield BLUEs from 290 tetraploid clones and the filtered set of SNPs, as described in the Section 2, were deployed for performing GWAS. Combining results from all GWAS models examined in the study revealed tuber yield QTL on all but two (7 and 9) chromosomes (Table S1, Figure 4, Figure S4) exhibiting polygenic control of the trait. One of the QTL hotspots for tuber yield was observed at the top end of chromosome 4 in the region spanning 1.6–7.1 Mb with two significant associations also detected towards the bottom end (Table 2). The closest significant association (solcap_snp_c2_11571) to *HSc70* was observed at 7.1 Mb that is, only 0.69 Mb away from the gene—emphasising the importance of this region in regulating tuber yield potential in a broad diverse tetraploid potato panel. It is also to be noted that the Infinium 8k Potato SNP Array (Felcher et al., 2012; Hamilton et al., 2011) employed in the current study covers only 3591 of the 39,031 protein coding genes (Potato Genome Sequencing Consortium, 2011) with a marker density of only 365 SNPs per Mb in the genic regions covered. Saturating the identified QTL hotspot region on chromosome 4 with highly dense markers, possibly sequencing the entire region, could potentially reveal an even closer or direct association with *HSc70*. Thus, there is a tuber yield QTL in the tetraploid association panel in the same region on chromosome 4 as that identified in the diploid biparental 06H1 population, emphasising the prevalence and importance of this locus in the genetic control of tuber yield.

**TABLE 2:** Tuber yield GWAS significant marker‐trait associations (MTAs) on chromosome 4

Marker^a^	Chromosome	Position (bp)	Genetic Model	GWAS model	Threshold^b^ (K, QK)	"‐log_10_ *p*" (K, QK)
Solcap_snp_c2_54382	4	1560109	General	K, QK	3.24, 3.19	3.94, 3.69
Solcap_snp_c1_15106	4	6866308	Diplo‐general	K, QK	3, 3.04	3.81, 3.52
Solcap_snp_c2_11571	4	7138123	Diplo‐general	K, QK	3, 3.04	3.56, 3.38
Solcap_snp_c2_43748	4	61552296	Diplo‐general	K, QK	3, 3.04	3.56, 3.37
Solcap_snp_c1_6188	4	64877602	Diplo‐general	K, QK	3, 3.04	3.01, 3.13

^a^ Results shown for genetic models consistent over K and QK GWAS models.

^b^ p‐value significance threshold, to control the genome‐wide false‐positive rate, established according to FDR method at 10% alpha level.

### 3.4 *HSc70* expression protects against photooxidative damage

**FIGURE 5:**
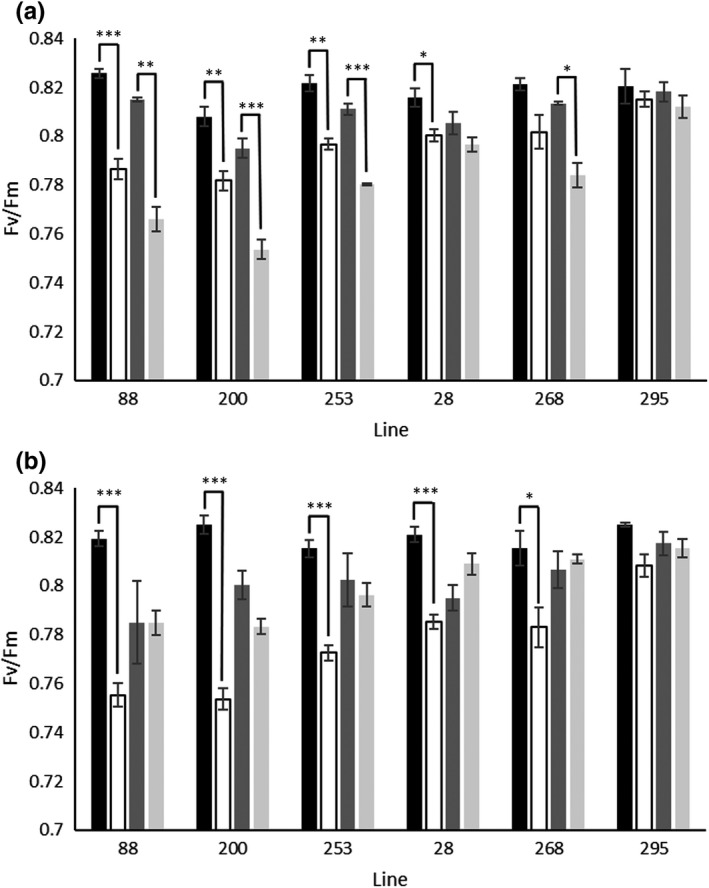
Impact of withholding water on 06H1 lines containing different *HSc70* alleles. Plants were acclimated to 18/15°C (A) or 30/24°C (B) day/night temperature. Plants were subjected to 3 days water withholding (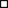) and then re‐watered for 4 days (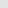) to determine recovery. Maximal quantum of yield of PSII (Fv/Fm) was determined in treated or age‐matched control plants (3 days 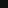, 7 days 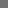) that were fully watered throughout the experiment. Data are represented as mean values ± SE, *n* = 3. Asterisks denote significant differences between treated and matched control samples (*p < 0.05; **p < 0.01; ***p < 0.005)

To define the impact of different *HSc70* alleles on short‐term plant stress responses under defined growth chamber conditions, three 06H1 lines containing the A1/A3 allelic combination (88, 200 and 253) and three lines containing the A2/A3 combination (28, 268 and 295) were subjected to drought and re‐watering under a moderate (18/15°C, day/night) and a high (30/24°C, day/night) temperature regime. The aim was to understand the impact that different *HSc70* alleles may have under inconsistent temperature and water availability as may be experienced under typical cultivation conditions. Following a period of water withholding for three days, all of the lines carrying the A1/A3 allelic combination exhibited a highly significant reduction in maximum quantum yield of PSII indicative of abiotic stress‐induced photosystem damage (Figure 5). This reduction was observed in these genotypes irrespective of the temperature regime and was greater at the higher temperature. In the A2/A3 genotypes, the damage was less consistent with only line 28 exhibiting a weakly significant (*p* < 0.05) reduction at the lower temperature (Figure 5a) and only two of the three genotypes showing a significant reduction at the higher temperature (Figure 5b). Unexpectedly, the higher temperature regime apparently promoted PSII repair following re‐watering where all genotypes exhibited no significant difference in Fv/Fm following 4 days re‐watering (Figure 5b). On the contrary, A1/A3 genotypes all continued to exhibit symptoms of damaged photosystems after 4 days of re‐watering at 18/15°C and line 268 carrying the A2/A3 allelic combination also exhibited a significant reduction in PSII quantum yield at this time‐point despite exhibiting no significant symptoms immediately after water withholding for 3 days.

Taken together these data suggest that the *HSc70* A2 allele provides protection against photooxidative damage to PSII induced by drought stress either alone or in combination with elevated temperature. It is likely that over a growing season, the ability to maintain or rapidly recover photosynthesis during or following intermittent stresses combined with a reduced resource requirement for photosystem repair supports increased yield and biomass.

### 3.5 *HSc70* expression in wild potato species

**FIGURE 6:**
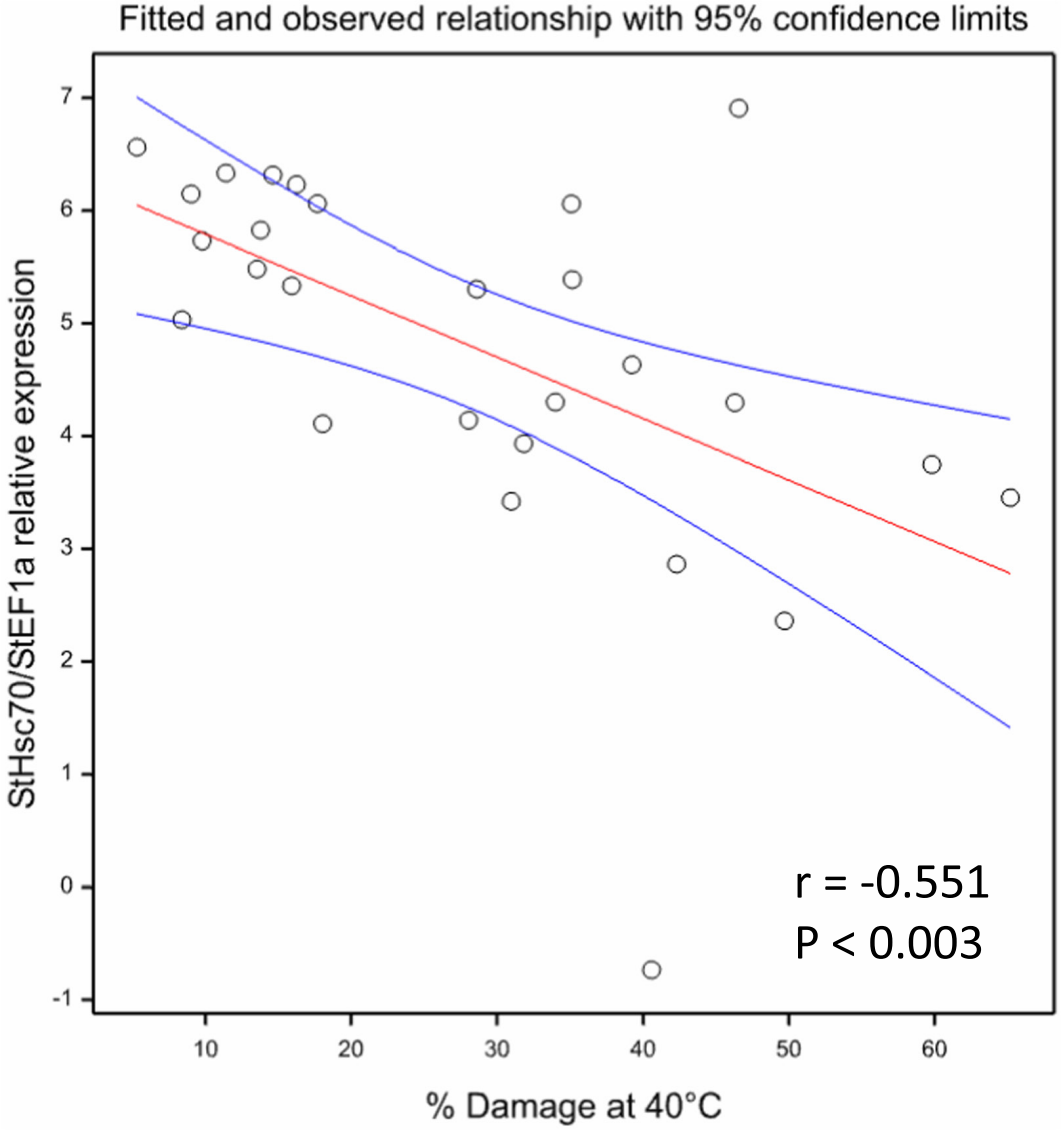
Simple linear regression of the relationship between Hsc70 expression and leaf damage in 26 wild potato accessions after 24 h at 40°C. Each data point represents a different accession. The blue lines represent the 95% confidence limit. y = 6.335+ −0.0545x, *R* ^2^ = 0.276, *p* < 0.003

Previously we identified significant variation in heat stress responses in wild potato species (Trapero‐Mozos, Ducreux, et al., 2018). Here, we investigated the relationship between *HSc70* expression level and heat tolerance in 26 wild potato accessions representing 23 wild species. As an indicator of heat tolerance, we assessed leaf membrane damage on exposure to elevated temperature with an electrolyte leakage assay (Trapero‐Mozos, Ducreux, et al., 2018) and the data presented in Figure S5. By simple linear regression, a highly significant, negative correlation (Pearson's correlation coefficient of −0.551, *p* < 0.003) between leaf membrane damage and *HSc70* expression was observed (Figure 6). The *R* ^2^ value was 0.276, thus 27.6% of the variation in leaf damage at 40°C can be explained by the regression model attributed to *HSc70* expression level.

The coding and promoter regions of the *HSc70* gene was sequenced in subset of 13 wild accessions to investigate whether any sequence elements correlated with heat‐induced expression level, as was seen for the A2 allele in the 06H1 population (Trapero‐Mozos, Morris, et al., 2018), where a repeated TA sequence in the promoter was demonstrated to impact on expression level. Promoter sequences representing 22 alleles from 13 species are aligned in Figure S6. These indicate that of the 13 diploid species accessions sampled, 4 are homozygous for the *HSc70* gene and 9 are heterozygous.

Alleles containing extended TA promoter sequences were present in two species originating from Western Mexico (namely, *S. jamesii* (JAM) (6 repeats) and *S. pinnatisectum* (PNT) (12 repeats), Figure S6,). These alleles were expressed at high relative levels on exposure to elevated temperature. However, some other alleles that contained fewer TA repeats were also strongly induced by heat (e.g. those from *S. raphanifolium* (RAP), *S. agrimonifolium* (AGF) and *S. verrucosum* (VER), all with 4 repeats). This might indicate other sequence elements can also modulate the expression levels of different *HSc70* alleles. In silico analysis of the ca. 1 kB of the 5′ DNA upstream sequence of the *Hsc70* promoter in 13 wild species indicated that these sequences possess the general characteristics of a promoter with a putative TATA box and CAAT box. (Figure S6). Sixteen transcription factor binding sites (TFBS) across all sequences were identified from NSITE‐PL (RegSite Plant DB, Softberry Inc., Mount Kisco, NY) and four of these TFBS were present in 12 out of 13 wild species (Figure S7). Several TFBS were present in only one species.

**FIGURE 7:**
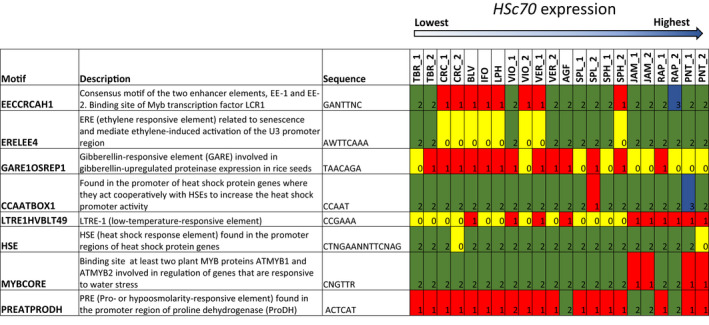
Occurrence of promoter elements upstream of the *HSc70* gene in a range of potato wild species

Plant cis‐acting regulatory DNA elements were identified from the PLACE database (Higo et al., 1999). As expected, elements responsive to hormones, stress, development, physiology, organ‐specific gene expression and defence were detected. A total of between 150 and 174 elements were predicted for each promoter sequence and are listed (Figure S7). A subset, reportedly related to abiotic stress or that vary in number or occurrence when related to *HSsc70* expression are presented in Figure 7. Of particular interest are a HSE heat shock response element where two copies were present in all species except PNT_allele_2 and CRC_allele_2 where they are missing. Additionally, a CCAATBOX1 element located immediately upstream from the most distal HSE of the promoter; ‘CCAAT box’ acts cooperatively with HSEs to increase promoter activity (3 copies in PNT_allele_1, 1 copy in SPL_allele_2 and two copies in all other alleles).

Interestingly, the copy number of four elements was associated with the level of *HSc70* expression. These include ERELEE4 where two copies were associated with high expression level (TBR outlier) and was absent when expression level was low; EECCRCAH1 with two copies generally associated with high expression and one copy in lower expressed accessions; GARE1OSREP1 absent in accessions with high expression level with a single copy present in lower; LTRE1HVBLT49—generally present in high expressed accessions JAM, RAP, PNT, AGF, VER, VIO absent in alleles from other accessions.

The deduced amino acid sequences of all alleles were very highly conserved with no obvious sequence variation correlating with electrolyte leakage data (Figure S8). These data strongly suggest an inverse relationship between *HSc70* expression level and cell damage on exposure to elevated temperature. Promoter elements that might be associated with temperature‐induced expression of *HSc70* were identified.

## 4 DISCUSSION

### 4.1 Field trials confirm *HSc70* alleles impact on tuber yield

By genetic analysis of a diploid potato population and a model tuberization system, we previously identified a *HSc70* gene on chromosome 4 as a candidate for conferring resilience of tuber yield under elevated temperature growth conditions (Trapero‐Mozos, Morris, et al., 2018). Although our previous transgenic studies support HSc70 as having a role in maintaining yield at elevated temperature, here we aimed to test genotypes with different allelic combinations of the *HSc70* gene under diverse field conditions. Genotypes were grown under much warmer and drier conditions than in the UK, at sites in Malawi and Kenya (Table 1).

The yield data from both African field trial sites showed a consistent pattern across two seasons. As with the screen from the previously reported model tuberization system, genotypes with the A2/A3 allelic combination were those that produced the highest tuber yield.

Analysis of tuber yield data from UK trials of the 06H1 population identified several tuber yield QTL including a QTL in the same region as the *HSc70* allele on chromosome 4 (Figure 2). As with field trials in Africa, in the UK trials the A1/A3 allelic combination was associated with the lowest tuber yield and the A2/A3 combination, the highest tuber yield. Deploying a panel of 290 tetraploid varieties, a GWAS analysis also detected a QTL in the *HSc70* region (Figure 4). We conclude that the field trials conducted in Africa validate the *HSc70* gene identified in an in vitro screen. Additionally, two independent approaches involving UK field trials of a diploid biparental mapping population (06H1) and a diverse tetraploid association panel also indicate that the same chromosomal region impacts on tuber yield, further implicating *HSc70* in tuber yield protection.

### 4.2 Physiological basis of yield effects

We hypothesise that *HSc70* expression provides protection against temperature‐induced cellular damage and translates to improved tuber yield over the course of a growing season. In order to investigate potential physiological mechanisms that explain *HSc70* impact on yield, we conducted controlled environment experiments where we introduced a defined drought stress under optimal or elevated temperatures and recorded stress‐induced changes in the maximal quantum yield of PSII (ɸ_PSII max_). Genotypes containing the A1/A3 allelic combination exhibited a more consistent and severe reduction in ɸ_PSII max_ than genotypes carrying the A2/A3 allele following drought stress and at the lower temperature also exhibited slower recovery following re‐watering. Such reduction in dark‐adapted leaves is normally interpreted as damage to the stress sensitive D1 protein of PSII (Murchie & Lawson, 2013) where drought‐induced reduction in stomatal conductance resulting in limited CO_2_ availability at the Rubisco active site can result in over‐reduction of the photosynthetic electron transport chain and oxidative turnover of D1 (Gururani et al., 2015). This interpretation was further supported by the observation that recovery of (ɸ_PSII max_) following re‐watering was promoted at the higher temperature where the synthesis of D1 protein has been previously demonstrated to be enhanced by moderately elevated temperatures (Miyata et al., 2015; Ueno et al., 2016).

### 4.3 Analysis of potato wild potato relatives for *HSc70* alleles

Potato wild relatives have evolved in a wide range of agro‐ecological niches and differential responses of tuber yield to elevated temperature have been reported (e.g. Guedes et al., 2019). Previously we applied an electrolyte leakage assay to indicate levels of heat‐induced membrane damage in leaves. From this assay, we showed that temperature‐induced leaf membrane damage was lower when expression levels of *HSc70* were higher in transgenic Desiree lines over‐expressing *HSc70*.

We also identified accessions of wild potato relatives that showed differential responses to elevated temperature based on the electrolyte leakage assay. Here, we have extended this analysis to show that in a wider selection of potato wild relatives, there was an inverse correlation between *HSc70* expression level and membrane damage that was highly significant. Previously we demonstrated that *HSc70* expression level was enhanced by a promoter TA repeat element (Trapero‐Mozos, Morris, et al., 2018) and so we cloned the *HSc70* alleles from the 13 wild potato accessions. Sequence analysis identified that two heat tolerant accessions originating from West Mexico (*S. jamesii* and *S. pinnatisectum*) contained at least one allele with a long run of TA repeats (up to 12 in the case of *S. pinnatisectum*). These alleles were also expressed at high levels under elevated temperature and extend our earlier findings about the role of TA repeats. However, several accessions did not have alleles with long TA repeats and yet also expressed *HSc70* at high levels and thus exhibited heat tolerance in the electrolyte leakage assay. This indicates that the TA repeat element is not the only factor that controls *HSc70* expression level. Our analysis indicated that in general the presence of the ERELEE4, EECCRCAH1 and LTRE1HVBLT49 elements is associated with high expression levels and the GARE1OSREP1 element is associated with low expression level.

The deduced amino acid sequences encoded by all the alleles that were examined were very highly conserved with no sequence variants associated with electrolyte leakage values. This suggests that the expression level of the *HSc70* gene underpins variation in heat protection.

## 5 CONCLUSIONS

In this paper, we present evidence supporting our hypothesis that a genetic factor controlling tuber yield under elevated temperature, identified using a tuberization model system was also effective in field trials conducted over several seasons and locations in warm dry environments in Kenya and Malawi. QTL for tuber yield in the *HSc70* region were also identified from trials conducted in the UK deploying both a biparental diploid population and a tetraploid GWAS panel, supporting our hypothesis about the importance to tuber yield of this locus. In experiments with wild potato species, we extend our observations and show that *HSc70* alleles that are highly expressed in response to elevated temperature, protect against heat‐induced damage. Genotypes carrying the *HSc70* A2 allele are protected against photooxidative damage to PSII induced by abiotic stresses. A TA repeat element in the *HSc70* promoter is associated with elevated *HSc70* expression although some genotypes have other mechanisms for achieving high levels of *HSc70* expression. Follow on experiments could explore the deployment of beneficial alleles of *HSc70* in breeding programmes via marker development.

## CONFLICT OF INTEREST

The authors declare that there is no conflict of interest.

## Supporting information

Fig S1

Fig S2

Fig S3

Fig S4

Fig S5

Fig S6

Fig S7

Fig S8

Table S1

Table S2

Supplementary Material
