# Supplementary figures and images for: Allelic variants of a potato HEAT SHOCK COGNATE 70 gene confer improved tuber yield under a wide range of environmental conditions
Source: Food Energy Secur. 2022 Mar 15;12(1):e377. doi: 10.1002/fes3.377 (PMC10078605; doi:10.1002/fes3.377)

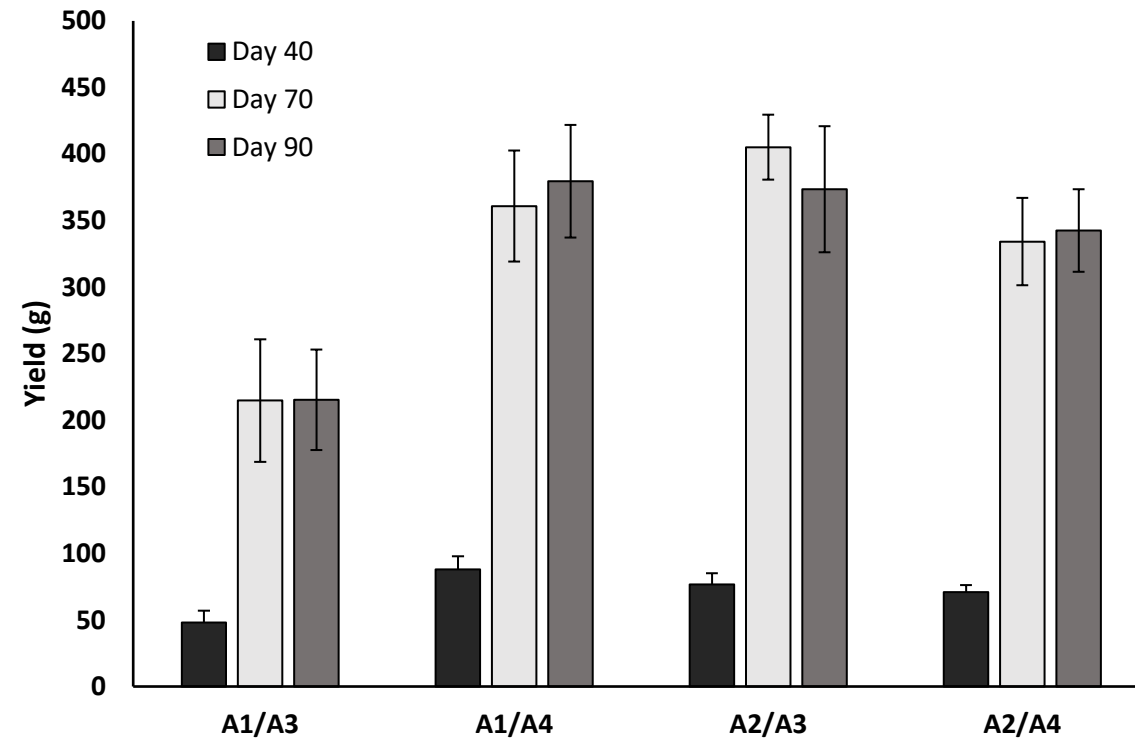

Supplement: Supplementary file 1 — Fig S1 [file FES3-12-0-s002.pdf]

**A**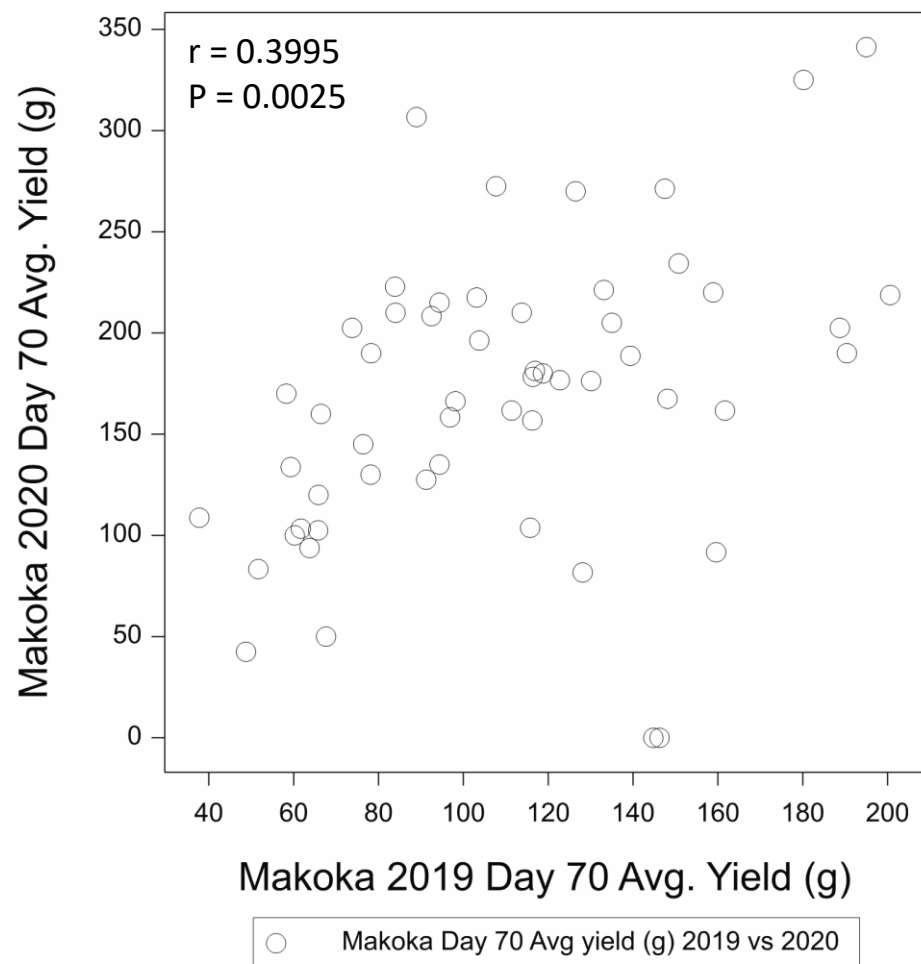**B**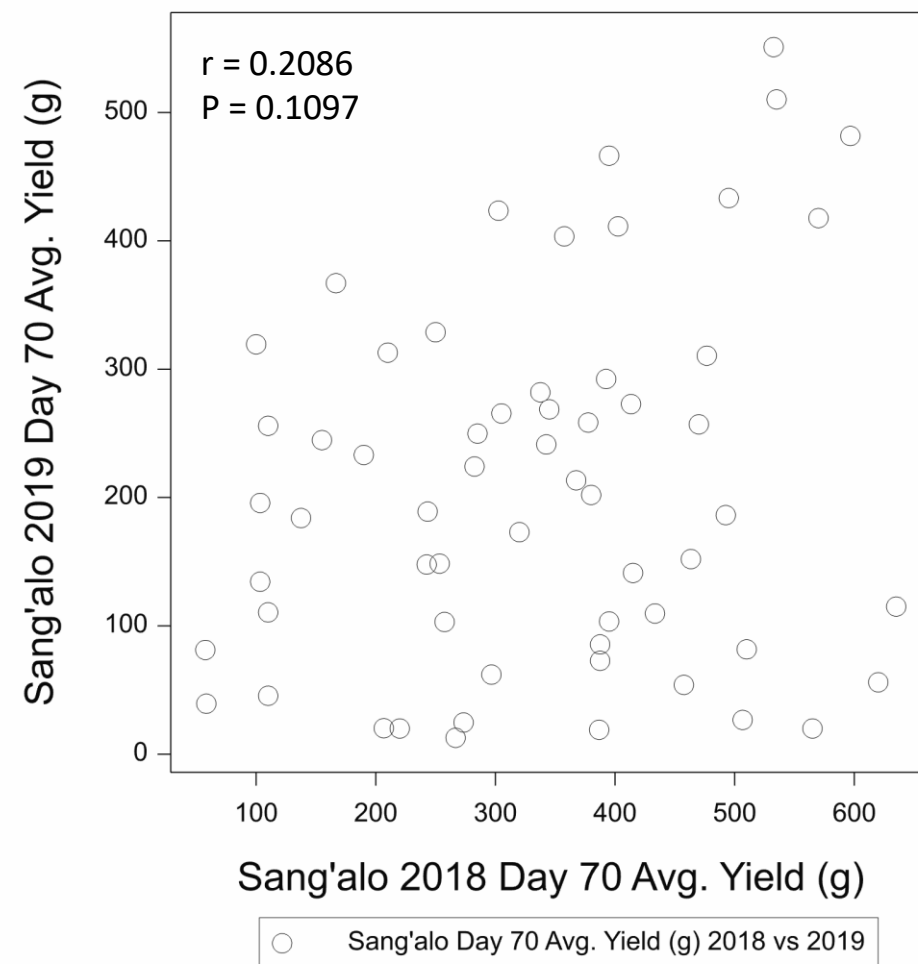

Supplement: Supplementary file 2 — Fig S2 [file FES3-12-0-s006.pdf]

**A**

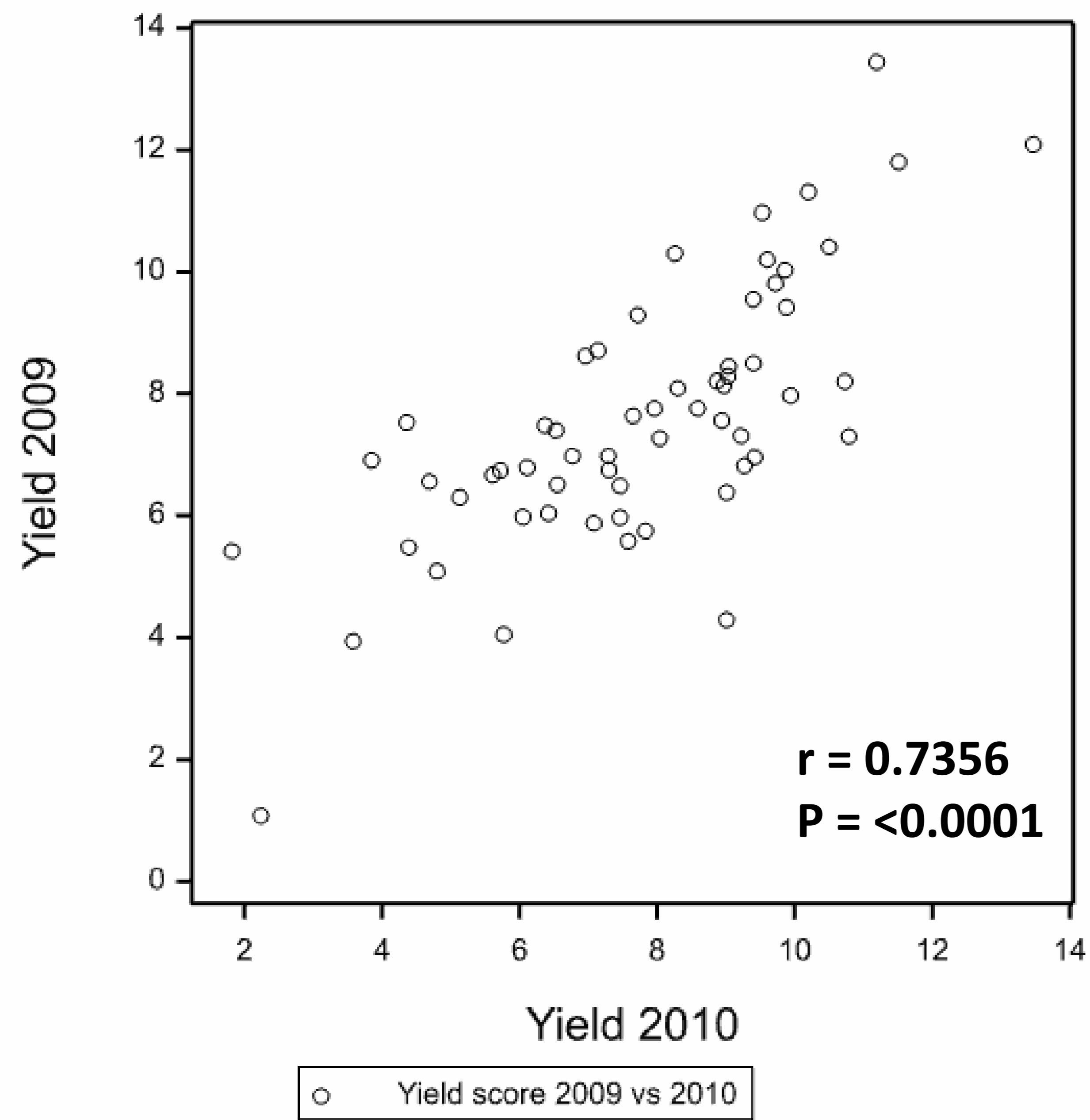

**B**

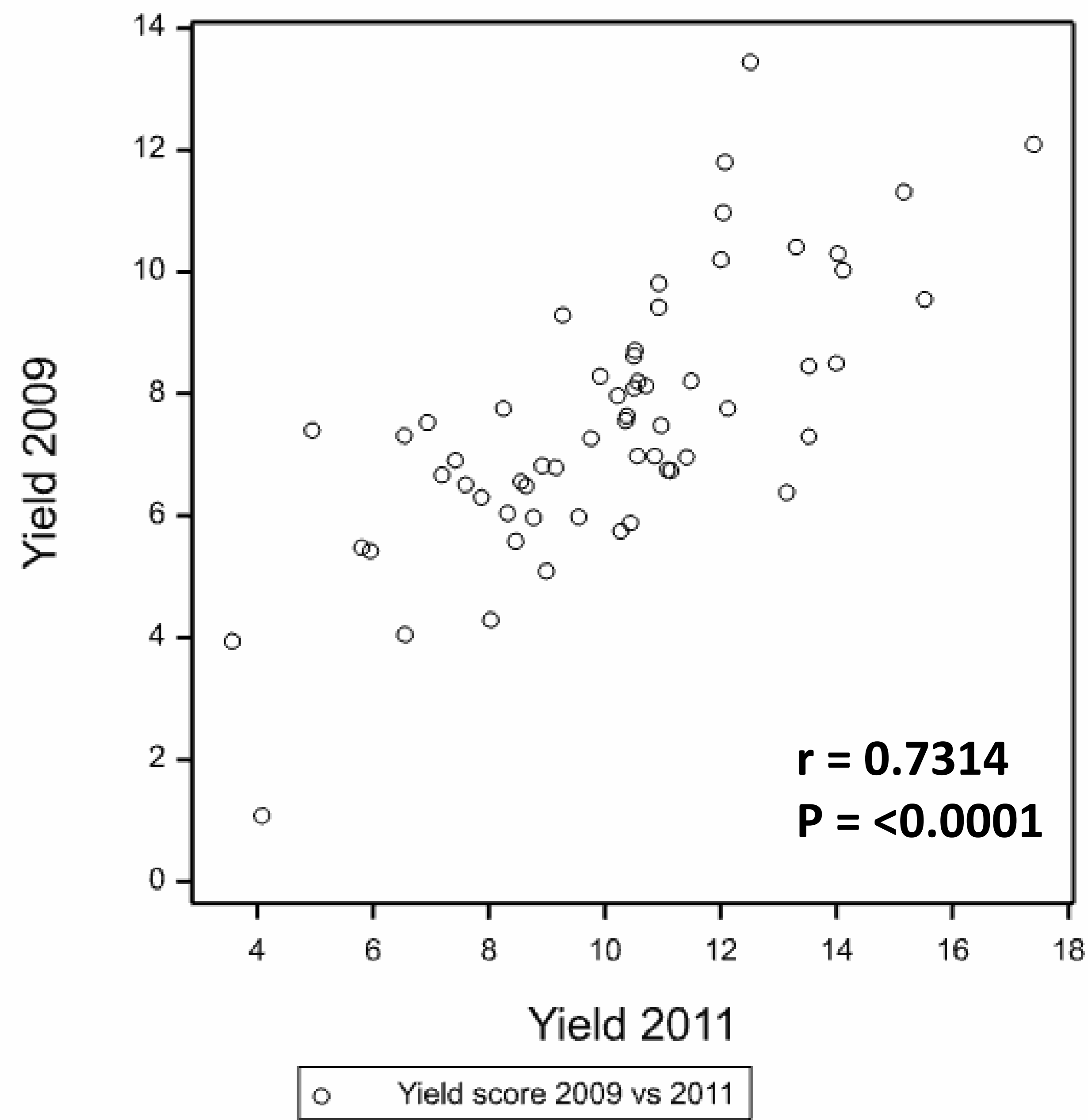

**C**

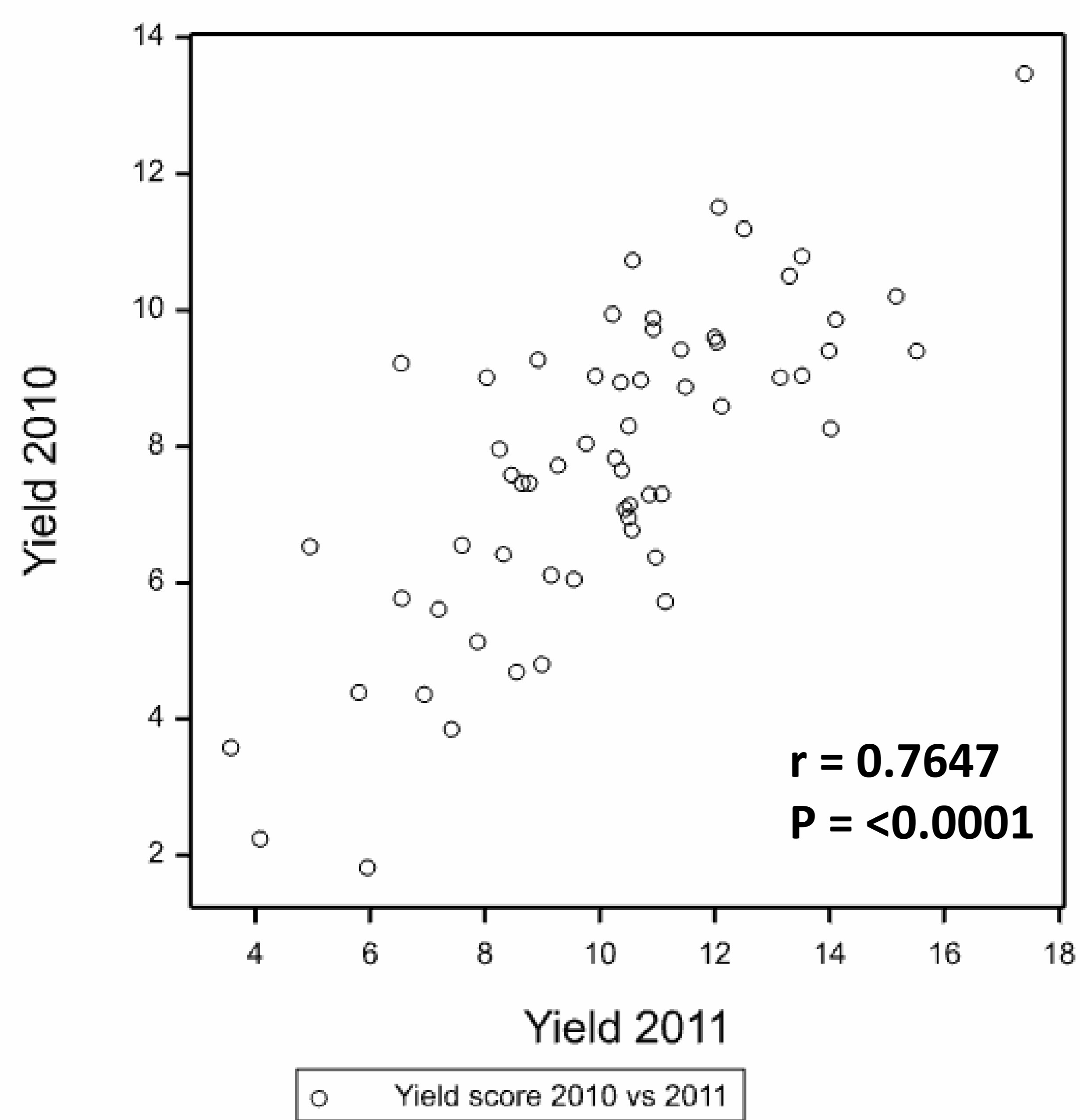

Supplement: Supplementary file 3 — Fig S3 [file FES3-12-0-s004.pdf]

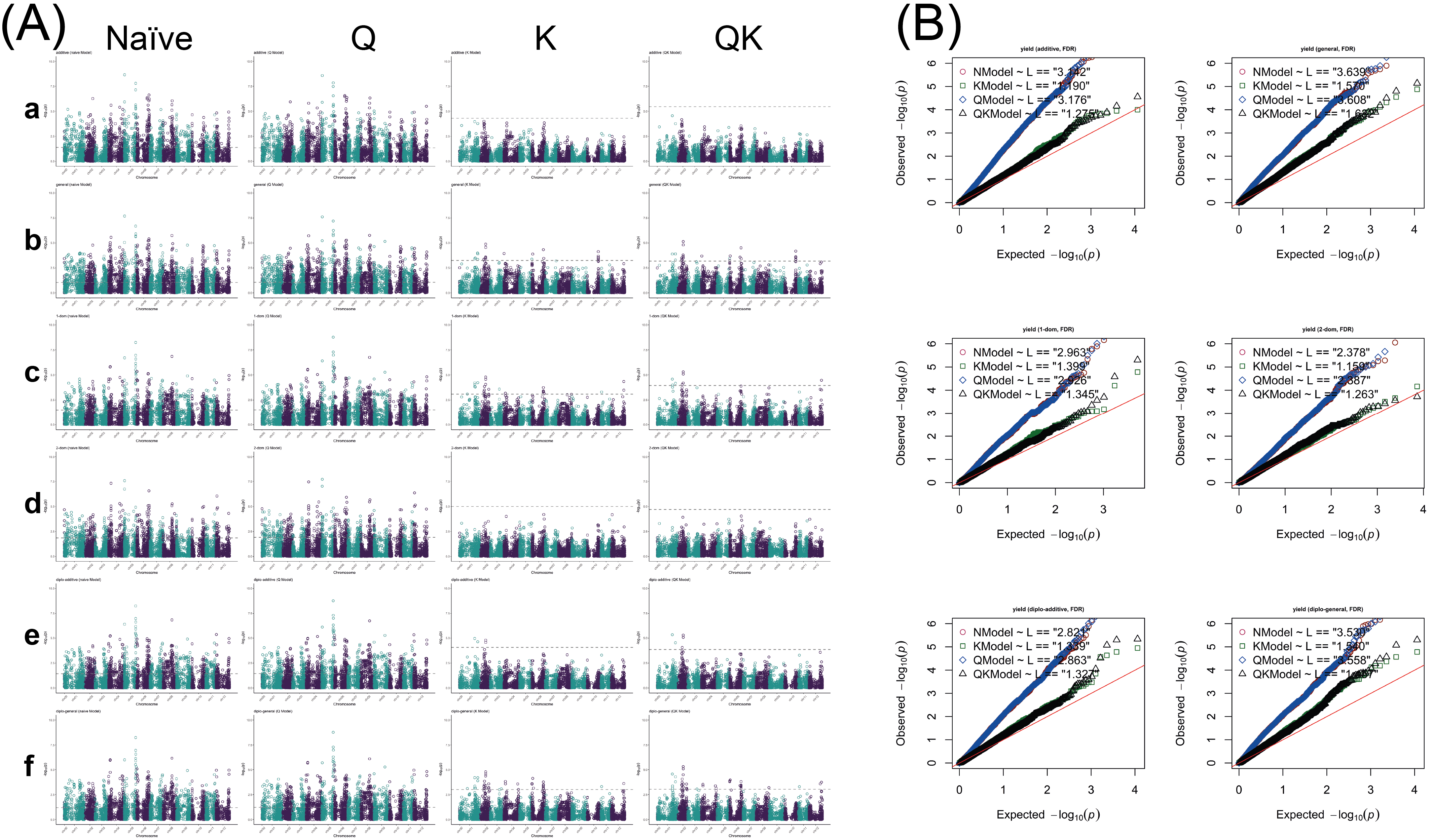

Supplement: Supplementary file 4 — Fig S4 [file FES3-12-0-s003.png]
