# Supplementary material for: Allelic variants of a potato HEAT SHOCK COGNATE 70 gene confer improved tuber yield under a wide range of environmental conditions
Source: Food Energy Secur. 2022 Mar 15;12(1):e377. doi: 10.1002/fes3.377 (PMC10078605; doi:10.1002/fes3.377)
Supplement: Supplementary file 5 — Fig S5 [file FES3-12-0-s007.pptx]

## Slide 1
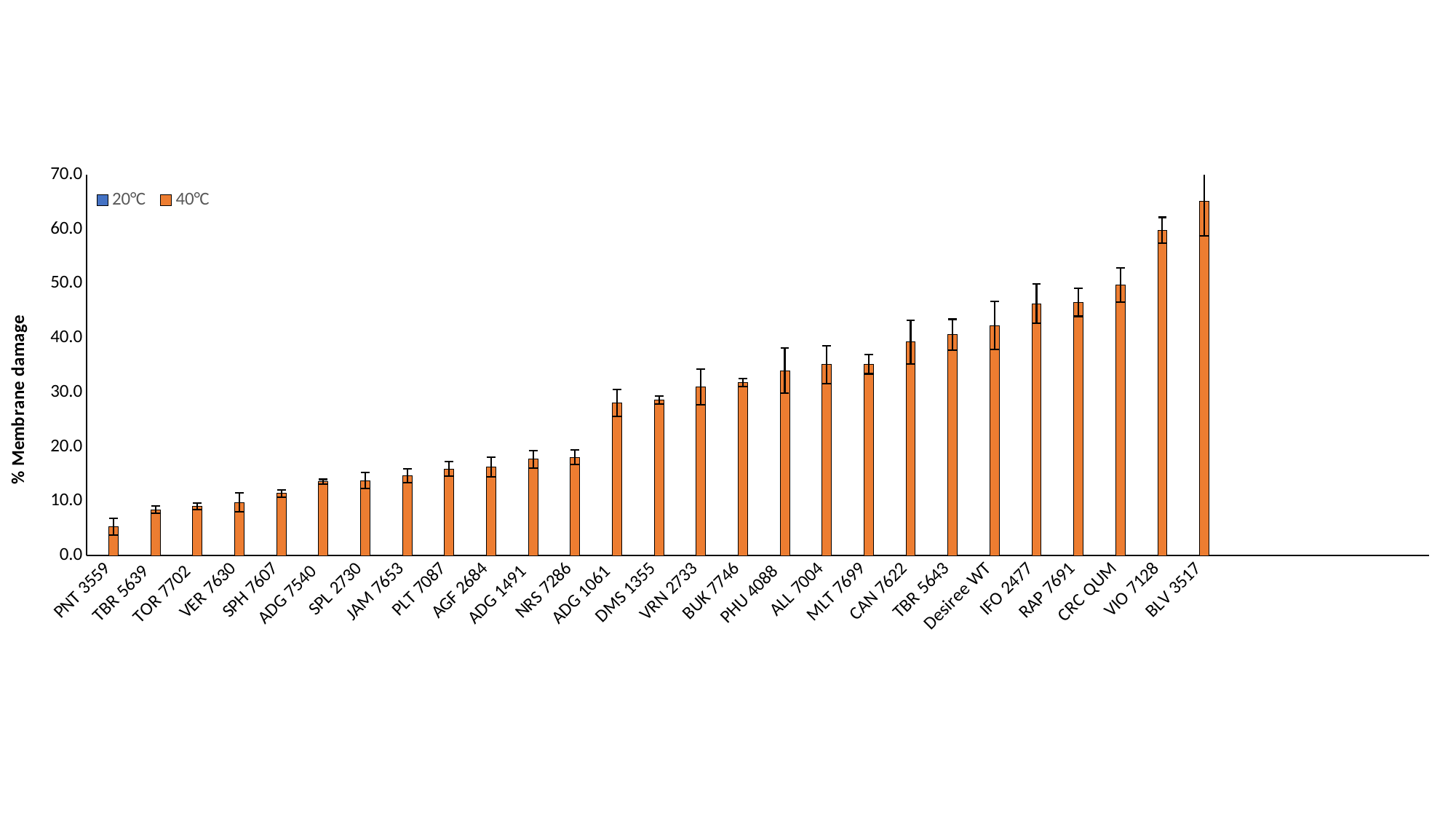

### Chart
| Category | 20°C | 40°C |
|---|---|---|
| PNT 3559 | 4.1 | 5.316974382912963 |
| TBR 5639 | 4.315923128383229 | 8.40306488906028 |
| TOR 7702 | 5.9279879896021965 | 9.014574354671575 |
| VER 7630 | 7.956946257579398 | 9.767534097110028 |
| SPH 7607 | 9.2 | 11.4 |
| ADG 7540 | 4.973915258494984 | 13.549866378745772 |
| SPL 2730 | 6.778791873688643 | 13.78975258952722 |
| JAM 7653 | 11.549999999999999 | 14.63 |
| PLT 7087 | 4.902983849806611 | 15.921036134543709 |
| AGF 2684 | 4.678224085120432 | 16.2431965320077 |
| ADG 1491 | 5.617160701998031 | 17.692932849255133 |
| NRS 7286 | 4.40791112317411 | 18.049646260172572 |
| ADG 1061 | 5.2550852823571566 | 28.048392085501714 |
| DMS 1355 | 4.09930783070529 | 28.597322869534366 |
| VRN 2733 | 4.5029770174443655 | 30.97164262215557 |
| BUK 7746 | 5.292867198397932 | 31.828562657206852 |
| PHU 4088 | 4.40707113035189 | 33.99607957739765 |
| ALL 7004 | 4.743074633216103 | 35.10599899281815 |
| MLT 7699 | 3.8244291185886907 | 35.16512840283332 |
| CAN 7622 | 4.317380660954711 | 39.240041928721176 |
| TBR 5643 | 5.164487098031428 | 40.584132071808405 |
| Desiree WT | 4.760136780079906 | 42.305985843963576 |
| IFO 2477 | 10.25 | 46.3 |
| RAP 7691 | 4.70403666274202 | 46.56852422048965 |
| CRC QUM | 6.54212818074501 | 49.70954579632371 |
| VIO 7128 | 6.9135 | 59.8136 |
| BLV 3517 | 9.8 | 65.2 |
