# Supplementary material for: Allelic variants of a potato HEAT SHOCK COGNATE 70 gene confer improved tuber yield under a wide range of environmental conditions
Source: Food Energy Secur. 2022 Mar 15;12(1):e377. doi: 10.1002/fes3.377 (PMC10078605; doi:10.1002/fes3.377)
Supplement: Supplementary file 6 — Fig S6 [file FES3-12-0-s009.docx]

**Figure S1** CLUSTAL O(1.2.4) multiple sequence alignment of CPC promoter sequence. The TA rep

PNT_allele_1 GTATAAACCAACATCTTGGATTTAACGAAGTGAGTTTATCATTTGAAATTCAAAATTGTT 60

JAM_allele_2 GTATAAACCAACATCTTGGATTTAACGAAGTGAGTTTATCATTTGAAATTCAAAATTGTT 60

JAM_allele_1 GTATAAACCAACATCTTGGATTTAACGAAGTGAGTTTATCATTTGAAATTCAAAATTGTT 60

VER_allele_1 GTATAAACCAACATCTTGGAATTAACTAAGTGAGTTTATCATTTGAAATTCAAAATTGTT 60

CRCQUM_allele_2 GTATAAACCAACATCTTGGAATTAACTAGGTGAGTTTATCATTTGACATTCAAAATTGTT 60

IFO GTATAAATCAACATCTTGGAATTAACTAAGTGAGTTTATCATTTGACATTCAAAATTGTT 60

BlV GTATAAACCAACATCTTGGAATTAACTAAGTGAGTTTATCATTTGACATTCAAAATTGTT 60

PNT_allele_2 GTATAAACCAACATCTTGGATTTAACGAAGTGAGTTTATCATTTGAAATTCAAAATTGTT 60

LPH GTATAAACCAACATCTTGGAATTAACTAAGTGAGTTTATCATTTGACATTCAAAATTGTT 60

VER_allele_2 GTATAAACCAACATCTTGGAATTAACTAAGTGAGTTTATCATTTGACATTCAAAATTGTT 60

AGF GTATAAACCAACATCTTGGAATTAACAAAATGAGTTTATCATTTGAAATTCAAAATTGTT 60

Vio_allele_1 GTATAAACCAACATCTTGGAATTAACAAAATGAGTTTATCATTTGAAATTCAAAATTGTT 60

CRCQUM_allele_1 GTATAAACCAACATCTTGGAATTAACTAAGTGAGTTTATCATTTGGCATTCAAAATTATT 60

Vio_allele_2 GTATAAACCAACATCTTGGAATTAACTAAGTGAGTTTATCATTTGACATTCAAAATTGTT 60

SPH_allele_2 GTATAAACCAACATCTTGGAATTAACTAAGTGAGTTTATCATTTGACATTCAAAATTGTT 60

RAP_allele_2 GTATAAACCAACATCTTGGAATTAACAAAATGAGTTTATCATTTGAAATTCAAAATTATT 60

RAP_allele_1 GTATAAACCAACATCTTGGAATTAACAAAGTGAGTTTATCATTTGAAATTCAAAATTGTT 60

SPL_allele_2 GTATAAACCAACATCTTGGAATTAACAAAGTGAGTTTATCATTTGAAATTCAAAATTGTT 60

SPH_allele_1 GTATAAACCAACATCTTGGAATTAACAAAGTGAGTTTATCATTTGAAATTCAAAATTGTT 60

TBR_allele_2 GTATAAACCAACATCTTGGAATTAACAAAGTGAGTTTATCATTTGAAATTCAAAATTGTT 60

SPL_allele_1 GTATAAACCAACATCTTGGAATTAACAAAGTGAGTTTATCATTTGAAATTCAAAATTGTT 60

TBR_allele_1 GTATAAACCAACATCTTGGAATTAACAAAGTGAGTTTATCATTTGAAATTCAAAATTGTT 60

******* ************ ***** * *************** ********** **

PNT_allele_1 AACTAAAACAACTTTAAAATACTTTTATTAACATATAATATTGTCTATTATGAACAAAAT 120

JAM_allele_2 AACTAAAACAACTTTAAAATACTTTTATTAACATATAATATTGTCTATTATGAACAAAAT 120

JAM_allele_1 AACTAAAACAACTTTAAAATACTTTTATTAACRTATAATATTGTCTATTATGAACAAAAT 120

VER_allele_1 AACTAAAACAACTTTAAAATACTTTTAATAAGATATAATATTGTCTGTTATAAACAAAAT 120

CRCQUM_allele_2 AGCTAAAACAACTTTAAAATACTTTTAATAAGACATAATATTGTCTGTTATGAACAAAAT 120

IFO AGCTAAAACAACTTTAAAATACTTTTAATAAGATATAATATTGTCTGTTATGAACAAAAT 120

Blv AGCTAAAACAACTTTAAAATACTTTTAATAAGATCTAATATTGTCTGTTATGAACAAAAT 120

PNT_allele_2 AACTAAAACAACTTTAAAATACTTTTATTAACATATAATATTGTCTATTATGAACAAAAT 120

LPH AGCTAAAACAACTTTAAAATACTTTTAATAAGATATAATATTGTCTGTTATGAACAAAAA 120

VER_allele_2 AGCTAAAACAACTTTAAAATACTTTTAATAAGATATAATATTGTCTGTTATGAACAAAAT 120

AGF AACTAAAACAACTTTAAAATACTTTTACTAAGATATAATATTGTCTGTTATGAACAAATT 120

Vio_allele_1 AACTAAAACAACTTTAAAATACTTTTACTAAGATATAATATTGTCTGTTATGAACAAATT 120

CRCQUM_allele_1 AGCTAAAACAACTTTAAAATACTTTTAATACGATATAATATTGTCTGTTATGAACAAAAT 120

Vio_allele_2 AGCTAAAACAACTTTAAAATACTTTTAATAAGATATAATATTGTCCGTTATGAACAAAAT 120

SPH_allele_2 AGCTAAAACAACTTTAAAATACTTTTAATAAGATATAATATTGTCTGTTATGAACAAAAT 120

RAP_allele_2 AACTACAACAACTTTAAAATACTTTTAATAAGGTATAATATTGTCCGTTATGAACAAAAT 120

RAP_allele_1 AACTAAAACAACTTTAAAATACTTTTAATAAGGTATAATATTGTCTGTTATGAACAAAAT 120

SPL_allele_2 AACTAAAACAACTTTAAAATACTTTTAATAAGATATAATATTGTCTGTTATGAACAAAAT 120

SPH_allele_1 AACTAAAACAACTTTAAAATACTTTTAATAAGATATAATATTGTCCGTTATGAACAAAAT 120

TBR_allele_2 AACTAAAACAACTTTAAAATACTTTTAATAAGATATAATATTGTCCGTTATGAACAAAAT 120

SPL_allele_1 AACTAAAACAACTTTAAAATACTTTTAATAAGATATAATATTGTCCGTTATGAACAAAAT 120

TBR_allele_1 AACTAAAACAACTTTAAAATACTTTTAATAAGATATAATACTGTCTGTTATGAACAAAAT 120

* *** ********************* ** ***** **** **** ******

PNT_allele_1 CCACATAATTCTTTCGGAAAATATTTCACATCATTAAGAATATCATGCACATTATATATA 180

JAM_allele_2 CCACATAATTCTTTCGGAAAATATTTCACATCATTAAGAATATCATGCACATTATATGTA 180

JAM_allele_1 CCACATAATTCTTTCGGAAAATATTTCACATCATTAAGAATATCATGCACATTATATGTA 180

VER_allele_1 CCGCATAGTTCTTTCGAAAAATATTTCACATCATTAAGAATATCATGCACGTTATATGTA 180

CRCQUM_allele_2 CCGCATAATTCTTTTGGGAAATATTTCACCTCATTAAGAATATCATGCACATTATATGTA 180

IFO CCGCATAATTCTTTCTGAAAATATTTCACATCATTAAGAATATCATGCACATTATATGTA 180

Blv CCGCATAATTCTTTCGGAAAATATTTCACATCATTAAGAATATCATGCACATTATATGTA 180

PNT_allele_2 CCACATAATTCTTTCGGAAAATATTTCACATCATTAAGAATATCATGCACATTATATGTA 180

LPH GCGCATAACTCTTTTGGAAAATATTTCACATCATTAAGAATATCATGCACATTATATGTA 180

VER_allele_2 CCGCATAATTCTTTCGGAAAATATTTCACATCATTAAGAATATCATGCACATTATATGTA 180

AGF CCGCATAATTCTTTCGGAAAATATTTCACATCATTAAGAATATCATGCACATTATATGTA 180

Vio_allele_1 CCGCATAATTCTTTCGGAAAATATTTCACATCATTAAGAATATCATGCACATTATATGTA 180

CRCQUM_allele_1 CCGCATAATTCTTTTGGGAAATATTTCACCTCATTAAGAATATCATGCACATTATATGTA 180

Vio_allele_2 CCGCATAATTCTTTTGAAAAATATTTCACATCATTAAGAATATCATGCACATTATATGTA 180

SPH_allele_2 CCGCATAATTCTTTCAGAAAATATTTCACATCATTAATACACTCGA-------------- 166

RAP_allele_2 CCGCATAATCCTTTTGGAAAATATTTTACATCATTAAGAATATCATGCACATTATATGTA 180

RAP_allele_1 CTGCATAATTCTTTCGGAAAATATTTCACATCATTAAGAATATCATGCACATTATATGTA 180

SPL_allele_2 CCGCATAATTCTTTCAGAAAATATTTCACATCATTA------------------------ 156

SPH_allele_1 CCGCATAATTCTTTTGGAAAATATTTCACATCATTAAGAATATCATGCACATTATATGTA 180

TBR_allele_2 CCGCATAATTCTTTTGGAAAATATTTCACATCATTAAGAATATCATGCACATTATATGTA 180

SPL_allele_1 CCGCATAATTCTTTTGGAAAATATTTCACATCATTAAGAATATCATGCACATTATATGTA 180

TBR_allele_1 CCGCATAATTCTTTTGGAAAATA-TTCACATCATTTAGAATATCATGCACATTATATGTA 179

**** **** ***** ** ** *****

PNT_allele_1 TCACATTTTCCCTCAAATATCAATATAAATTTTGTTCAGACGCTCGACACAACTCAGTAT 240

JAM_allele_2 TCACATTTTCCCTCAAATATCAATATAAATTTTGTTCATACGCTCGACACAACTCAGTAT 240

JAM_allele_1 TCACATTTTCCCTCAAATATCAATATAAATTTTGTTCATACGCTCGACACAACTCAGTAT 240

VER_allele_1 TCACATTTTCCCTCAAATATTAATATAAATTTTGTTCATACGCTCGATACAAAATC-AGT 239

CRCQUM_allele_2 TCACATTTTCCCTCAAATATCAATATAAATTTTGTTTATACGCTCGACACAAATCAGTA- 239

IFO TCACATTTCCCCTCAAATATCAATATAAATTTTGTTCATACGCTCGACACAAATCAGTA- 239

Blv TCACATTTTCCCTCAAATATCAATATAAATTTTGTTCATACGCTCGACACAAATCAATA- 239

PNT_allele_2 TCACATTTCCCCTCAAATATCAATATAAATTTTGTTCATATGCTCGACAGAAATCAATA- 239

LPH TCACATTTCCCCTCAAATATCAATATAAATTTTGTTCATATGCTCGACAGAAATCAATA- 239

VER_allele_2 TCACATTTTCCCTCAAATATCAATATAAATTTTGTTCATACGCTCGATACAAATCAGTA- 239

AGF TCACATTTTCCCTCAAATATCAATATAAATTTTGTTCATACGCTCGACACAAATCAGTA- 239

Vio_allele_1 TCACATTTTCCCTCAAATATCAATATAAATTTTGTTCATACGCTCGACACAAATCAGTA- 239

CRCQUM_allele_1 TCACATTTTCCCTCAAATATCAATATAAATTTTGTTCATACGCTCGACATAAATCAATAT 240

Vio_allele_2 TCACATTTTCCCTCAAATATCAATATAAATTTTGTTCATACTCGACACAAATCAGTATA- 239

SPH_allele_2 ---------------------------------------------CACAAATCAATATA- 180

RAP_allele_2 TCACATTTTCCCTCAAATATCAATATAAATTTTGTTCATACGCTCGACACAAATCAGCA- 239

RAP_allele_1 TCACATTTTCTCTCAAATATCAATATAAACTTTGTTCATACGCTCGACACAAATCAGTA- 239

SPL_allele_2 -------------------------------------ATACACTCGACACAAATCAATA- 178

SPH_allele_1 TCACATTTTCCCTCAAATATCAATATAAATTTTGTTCATACTCTCGACACAAATCAGTA- 239

TBR_allele_2 TCACATTTTCCCTCAAATATCAATATAAATTTTGTTCATACTCTCGACACAAATCAGTA- 239

SPL_allele_1 TCACATTTTCCCTCAAATATCAATATAAATTTTGTTCATACTCTCGACACAAATCAGTA- 239

TBR_allele_1 TCACATTTTCCCTCAAATATCAATATAAATTTTGTTCATACTCTCGACACAAATCAGTA- 238

* * *

PNT_allele_1 ATATATATATATATATATATATATCTTGAATAATTAACTCAATTTTACCAGCTCACAAGT 300

JAM_allele_2 ATATATATA--------------TCTTGAATAATTAACTCAATTTTACCAGCTCACAAGT 286

JAM_allele_1 ATATATA----------------TCTTGAATAATTAACTCAATTTTACCAGCTCACAAGT 284

VER_allele_1 ATATATACA------------TATCTTGAATAATTAACTCAATTTTACAAGCTCA----- 282

CRCQUM_allele_2 -TATA----------------TATATTGAATAATTAACTCAATTTTACGAGCTCA----- 277

IFO -TATATACA------------TATCTTGAATAATTAACTCAATTTTACAAGCTCA----- 281

Blv -TATA----------------TATATTGAATAATTAACTCAATTTTACAAGCTCA----- 277

PNT_allele_2 -TTTATACA------------TATCTTGTATAATTAACTCAATTTTACAAGCTCA----- 281

LPH -TTTATACA------------TATCTTGTATAATTAACTCAATTTTACAAGCTCA----- 281

VER_allele_2 -TATATACA------------TATCTTGAATAATTAACTCAATTTTACAAGCTCA----- 281

AGF -TATATACA------------TATCTTGAATAATTAACTCAATTTTACAAGCTCA----- 281

Vio_allele_1 -TATATACA------------TATCTTGAATAATTAACTCAATTTTACAAGCTCA----- 281

CRCQUM_allele_1 ATATGTACA------------TATCTTGAATAATTAACTCAATTTTACAAGCTCA----- 283

Vio_allele_2 ------------------------------------TAATTATTTTACAAGCTCA----- 258

SPH_allele_2 -TATATACA------------TATCTTGAATAATTAATTCAATTTTACAAGCTCA----- 222

RAP_allele_2 -TATATACA------------CATCTTGAATAATTAATTCAATTTTACAAGCTCA----- 281

RAP_allele_1 -TATATACA------------TATATTGAATAATTAACTCAATTTTACAAGCTCA----- 281

SPL_allele_2 -TATATACA------------TATCTTGAATAATTAATTCAATTTTACAAGCTCA----- 220

SPH_allele_1 -TATATACA------------TATCTTGAATAATTAACTCAATTTTACAAGCTCA----- 281

TBR_allele_2 -TATATACA------------TATCTTGAATAATTAACTCAATTTTACAAGCTCA----- 281

SPL_allele_1 -TATATACA------------TATCTTGAATAATTAACTCAATTTTACAAGCTCA----- 281

TBR_allele_1 -TATATACA------------TATCTTGAATAATTAACTCAATTTTACAAGCTCA----- 280

******* ******

PNT_allele_1 CACAACATTTATTTTGAACGGACTAAC-ATGTTGAATTTAAAGTAAAGTTCAAAATTATT 359

JAM_allele_2 CACAACATTTATTTTGAACATATAAAC-ATGTTGAATTTAAAGTGAAGTTCAAAATTATT 345

JAM_allele_1 CACAACATTTATTTTGAACATATAAAC-ATGTTGAATTTAAAGTGAAGTTCAAAATTATT 343

VER_allele_1 --CAACATTTATTTTGAACGGACTAAC-ATGCCAAATTTAAAGTAAAGTTCAAAATTATT 339

CRCQUM_allele_2 --CAACATTTATTTTGAACGGACTAACAATGTTGAATTTAAAGTAAAGTTCAAAATTATT 335

IFO --CAACATTTATTTTGAACGGACTAAC-ATGTCAAATTTAAAGTAAAGTTCAAAATTATT 338

Blv --CAACATTTATTTTGAACGGACTAAC-ATGTTGAATTTAAAGTAAAGTTCAAAATTATT 334

PNT_allele_2 --CAACATTTATTTTGAACGGACTAAC-ATGTTGAATTTAAAGTAAAGTTCAAAATTATT 338

LPH --CAACATTTATTTTGAACGGACTAAC-ATGTTGAATTTAAAGTAAAGTTCAAAATTATT 338

VER_allele_2 --CAACATTTATTTTGAACAGAGTAAC-ATGTTGAATTTAAAGTAAAGTTCAAAATTATT 338

AGF --CAACATTTATTTTGAACGGGCTAAC-ATGTTGAATTTAAAGTAAAGTTCAAAATTATT 338

Vio_allele_1 --CAACATTTATTTTGAACGGGCTAAC-ATGTTGAATTTAAAGTAAAGTTCAAAATTATT 338

CRCQUM_allele_1 --CAACATTTATTTTGAACGGACTA----------ATTTAAAGTAAAGTTCAAAATTATT 331

Vio_allele_2 --CAACATTTATTTTGAATGGACTAAC-ATGTTGAATTTAAAGTAAAGTTCAAAATTATT 315

SPH_allele_2 --CAACATTTATTTTGAACGGACTAAC-ATGTTGAATTTAAAGTAAAGTTCAAAATTATT 279

RAP_allele_2 --CAACATTTATTTTGAACGGACTAAC-ATGTTGAATTTAAAGTAAAGTTCAAAATTATT 338

RAP_allele_1 --CAACATTTATTTTGAACGGACTAAC-ATGTCAAATTTAAAGTAAAGTTCAAAATTATT 338

SPL_allele_2 --CAACATTTATTTTGAACGGACTAAC-ATGTCAAATTTAAAGTAAAGTTCAAAATTATT 277

SPH_allele_1 --CAACATTTATTTTGAACGGACTAAC-ATGTTGAATTTAAAGTAAAGTTCAAAATTATT 338

TBR_allele_2 --CAACATTTATTTTGAACGGACTAAC-ATGTTGAATTTAAAGTAAAGTTCAAAATTATT 338

SPL_allele_1 --CAACATTTATTTTGAACGGACTAAC-ATGTTGAATTTAAAGTAAAGTTCAAAATTATT 338

TBR_allele_1 --CAACATTTATTTTGAACGGACTAAC-ATGTTGAATTTAAAGTAAAGTTCAAAATTATT 337

**************** * ********* ***************

PNT_allele_1 TTTATAGATTTAAATAAATTTAATATAATTAAATGCCTATATTAACATTTAAAATTAAAA 419

JAM_allele_2 TCTATAGATTTA----AATTTAATATAATTAAATGCCTACATTAACATTTAAAATTAAAA 401

JAM_allele_1 TCTATAGATTTA----AATTTAATATAATTAAATGCCTACATTAACATTTAAAATTAAAA 399

VER_allele_1 TTTATAGATTTA----AATTTAATATAATTAAATGCCTATATTAACATTTTAAATTAAAA 395

CRCQUM_allele_2 TTTATAGATTTA----AATTTAATATAATTAAATGCCTATATTAACATTTAAAATTAAAA 391

IFO TTTATAGATTTA----AATT-AATATAATTAAATGCCTATGTTAACATTTAAAATGAAAA 393

Blv TTTATAGGTTTA----AATTTAATATAATTAAATGCATATATTAACATTTAAAATTAAAA 390

PNT_allele_2 TTTATAGGTTTA----AATTTAATATAATTAAATGCTTATATTAACATTTAAAATTAAAA 394

LPH TTTATAGGTTTA----AATTTAATATAATTAAATGCTTATATTAACATTTAAAATTAAAA 394

VER_allele_2 TTTGTAGGTTTA----AATTTAATATAATTAAATGCATATATTAACATTTAAAATTAAAA 394

AGF TTTATAGGTTTA----AATTTAATATAATTAAATGCATATATTAACATTTAAAATTAAAA 394

Vio_allele_1 TTTATAGGTTTA----AATTTAATATAATTAAATGCATATATTAACATTTAAAATTAAAA 394

CRCQUM_allele_1 TTTATAGATTTA----AATTTAATATAATTAAATGCCTATATTAACATTTAAAATTAAAA 387

Vio_allele_2 TTCATAGATTTA----AAGTTAATATAATTAAATGCCTATATTAACATTTAAATTAAAAT 371

SPH_allele_2 TTTATAGATTTA----AA-TTAATACAATTAAATGCCTATATTAACATTTAAAATTAAAA 334

RAP_allele_2 TTTATAGATTTA----AA-TTAATATAATTAAATGCCTGTATTAACATTTAAAAATAAAA 393

RAP_allele_1 TTTATAGATTTA----AA-TTAATATAATTAAATGCCTGTATTAACATTTAAAAATAAAA 393

SPL_allele_2 TTTATAGATTTA----AA-TTAATATAATTAAATGCCTATATTAACATATAAAATTAAAA 332

SPH_allele_1 TTTATAGATTTA----AATTTAATATAATTAAATGCCTATATTAACATTTAAAATTAAAA 394

TBR_allele_2 TTTATAGATTTA----AATTTAATATAATTAAATGCCTATATTAACATTTAAAATTAAAA 394

SPL_allele_1 TTTATAGATTTA----AATTTAATATAATTAAATGCCTATATTAACATTTAAAATTAAAA 394

TBR_allele_1 TTTATAGATTTA----AATTTAATATAATTAAATGCCTATATTAACATTTAAAATTAAAA 393

* *** **** ** * **** ********** * ******* * ** ***

PNT_allele_1 TGTTACTAGTACTTACTACTTACTAGAACTTAATACTAGAGGAAAAAGTAAGAAAATTGA 479

JAM_allele_2 TGTTAGTAGTATTTACTACTTACTAGAACTTAATACTAGAGGAAAAAGTACGAAAATTGA 461

JAM_allele_1 TGTTAGTAGTATTTACTACTTACTAGAACTTAATACTAGAGGAAAAAGTACGAAAATTGA 459

VER_allele_1 TGTAGTACTAGT-----ACTTACTAGAACTTAATACTAGAGGAAAAAGTAAGAAAATTGA 450

CRCQUM_allele_2 CG---TTAGTAC-----TTACTAGAACTTATAATACTAGAGGAAAAAGTAAGAAAAATGA 443

IFO TGTTACTAGTAC-----TCACTAGAACTTATAATACTAGAGGAAAAAGTAAGAAAATTGA 448

Blv TG---TTAGTAC-----TTACTAGAACTTATAATACTAGAGGAAAAAGTAAGAAAATTGA 442

PNT_allele_2 TG---TTAGTAC-----TTACTAGAACTTATAATACTAGAGGAAAAAGTAAGAAAATTGA 446

LPH TG---TTAGTAC-----TTACTAGAACTTATAATACTAGAGGAAAAAGTAAGAAAATTGA 446

VER_allele_2 TG---TTAGTAC-----TTACTAGAACTTATAATACTAGAGGAAAAAGTAAGAAAATTGA 446

AGF T---------------------------------GTTAGAGGAAAAAGTAAGAAAATTGA 421

Vio_allele_1 T---------------------------------GTTAGAGGAAAAAGTAAGAAAATTGA 421

CRCQUM_allele_1 TGTTAGTACTTA-----CTAGAA-----CTTAATACTAGAGGAAAAAGTAAGAAAATTGA 437

Vio_allele_2 -G---TTAGTAC-----TTACTAGAACTTATAATACTAGAGGAAAATGTAAGAAAATTGA 422

SPH_allele_2 TGTTAGTACTTA-----C---TAGAACTTATAATACTAGAGGAAAAAGTAAGAAAATTGA 386

RAP_allele_2 TGTTACTAGTAC-----TTACTAGAACTTATAATACTAGAGGAAAAAGTAAGAAAATTGA 448

RAP_allele_1 TGTTACTAGTAC-----TTACTAGAACTTATAATACTAGAGGAAAAAGTAAGAAAATTGA 448

SPL_allele_2 TGTTACTAGTAC-----TTGCTAG-AACTTAAATACTAGAGGAAAAAGTAAGAAAATTGA 386

SPH_allele_1 TGTTAGTACTTA-----CTAGAAC-TTAT--AATACTAGAGGAAAAAGTAAGAAAATTGA 446

TBR_allele_2 TGTTAGTACTTA-----CTAGAAC-TTAT--AATACTAGAGGAAAAAGTAAGAAAATTGA 446

SPL_allele_1 TGTTAGTACTTA-----CTAGAAC-TTAT--AATACTAGAGGAAAAAGTAAGAAAATTGA 446

TBR_allele_1 TGTTAGTACTTA-----CTAGAAC-TTATATAATACTAGAGGAAAAAGTAAGAAAATTGA 447

********** *** ***** ***

PNT_allele_1 GCGAAACATTATGAAACACATTTAGAACTGTAGAGAAATCTCCAGAACTCACCAGAAGCT 539

JAM_allele_2 GCGAAACATTATGAAACAGATCTAGAACTGTAGAGAAATCTCCAGAACTTACCAGAAGCT 521

JAM_allele_1 GCGAAACATTATGAAACAGATCTAGAACTGTAGAGAAATCTCCAGAACTTACCAGAAGCT 519

VER_allele_1 GCGAAGCATTATGAAACACGTCTAGAACTGTAGAGAAATCTCCAGAACTTACCAGAAGCT 510

CRCQUM_allele_2 GCGAAGCATTATGAAACACGTCTAGAACTGTAGAGAAATCTCCAGAACTTACCAGAAGCT 503

IFO GCGAAGCATTATGAAACACGTCTAGGACTGTAGAGAAATCTCCAGAACTTACCAGAAGCT 508

Blv GCGAAGCATTATGAAACACGTCTAGAACTGTAGAGAAATCTCCAGAACTTACCAGAAGCT 502

PNT_allele_2 GCGAAGCATTATGAAACACGTCTAGAACTGTAGAGAAATCTCCAGAACTTACCAGAAGCT 506

LPH GCGAAGCATTATGAAACACGTCTAGAACTGTAGAGAAATCTCCAGAACTTACCAGAAGCT 506

VER_allele_2 GCGAAGCATTATGAAACACGTCTAGAACTGTAGAGAAATCTCCAGAACTTACCAGAAGCT 506

AGF GCGAAGCATTATGAAACACGTCTAGAACTGTAGAGAAATCTCCAGAACTTACCAGAAGCT 481

Vio_allele_1 GCGAAGCATTATGAAACACGTCTAGAACTGTAGAGAAATCTCCAGAACTTACCAGAAGCT 481

CRCQUM_allele_1 GCGAAGCATTATGAAACACGTCTAGAACTGTAGAGAAATCTCCAGAACTTACCAGAAGCT 497

Vio_allele_2 GCGAAGCATTATGAAACACGTCTAGAACTGTAGAGAAATCTCCAGAACTTACCAGAAGCT 482

SPH_allele_2 GCGAAGCATTATGAAACACGTCTAGAACTGTAGAGAAATCTCCAGAACTTACCAGAAGCT 446

RAP_allele_2 GCGAAGCATTATGAAACACGTCTAGACCTGTAGAGAAATCTCCAGAACTTACCAGAAGCT 508

RAP_allele_1 GCGAAGCATTATGAAACACGTCTAGAACTGTAGAGAAATCTCCAGAACTTACCAGAAGCT 508

SPL_allele_2 GCGAAGCATTATGAAACACGTCTAGAACTGTAGAGAAATCTCCAGAACTTACCAGAAGCT 446

SPH_allele_1 GCGAAGCATTATGAAACACGTCTAGAACTGTAGAGAAATCTCCAGAACTTACCAGAAGCT 506

TBR_allele_2 GCGAAGCATTATGAAACACGTCTAGAACTGTAGAGAAATCTCCAGAACTTACCAGAAGCT 506

SPL_allele_1 GCGAAGCATTATGAAACACGTCTAGAACTGTAGAGAAATCTCCAGAACTTACCAGAAGCT 506

TBR_allele_1 GCGAAGCATTATGAAACACGTCTAGAACTGTAGAGAAATCTCCAGAACTTACCAGAAGCT 507

***** ************ * *** ********************** **********

PNT_allele_1 ACAAGCAATTGGTCAAAAAA-AAAACCTTTCATCACCAAAACTCACCGTTTTGGTATCCA 598

JAM_allele_2 ACAAGCAATTGGTCAAAAAG-AAAACCTTTCATCACCAAAACTCACCGTTTTGGTATCCA 580

JAM_allele_1 ACAAGCAATTGGTCAAAAAG-AAAACCTTTCATCACCAAAACTCACCGTTTTGGTATCCA 578

VER_allele_1 ACAAGCAATTGGTCAAAAA--AAAAACTTTCATCACCAAAACTCACCGTTTTTGTATCCA 568

CRCQUM_allele_2 ACAAGCAATTGGTCAAACA--AAAAACTTTCATCACTAAAACTCACCGTTTTTGTATCCA 561

IFO ACAAGCAATTGGTCAAAAAA-AAAAACTTTCATCACCAAAACTCACCGTTTTTGTATCCA 567

Blv ACAAGCAATTGGTCAAAAA--AAAAACTTTCATCACCAAAACTCACCGTTTTTGTATCCA 560

PNT_allele_2 ACAAGCAATTGGTCAAAAA--AAAAACTTTCATCACCAAAACTCACCGTTTTTGTATCCA 564

LPH ACAAGCAATTGGTCAAAAA--AAAAACTTTCATCACCAAAACTCACCGTTTTTGTATCCA 564

VER_allele_2 ACAAGCGATTGGTCAAAAAA-AAAAACTTTCATCACCAAAACTCACCGTTTTTGTATCCA 565

AGF ACAAGCAATTGGTCAAAAAA--AAAACATTCATCACCAAAACTCACCGTTTTTGTATCCA 539

Vio_allele_1 ACAAGCAATTGGTCAAAAAA--AAAACATTCATCACCAAAACTCACCGTTTTTGTATCCA 539

CRCQUM_allele_1 ATAAGCAATTGGTTAAAAA--AAAAACTTTCATTACTAAAACTCACCGTTTTTGTATCCA 555

Vio_allele_2 ACAAGCAATTGGTCAAAAAAAAAAAACTTTTATCACCAAAACTCACCGTTTTTCTATCCA 542

SPH_allele_2 ACAAGCAATTGGTCAAAA---AAAATCTTTCATCACCAAAACTCACCGTTTTTGTATCCA 503

RAP_allele_2 ACAAGCAATTGGTCAAAAAAAAAAAACTTTCATCACCGAAACTCACCGTTTTTGTATCCA 568

RAP_allele_1 ACAAGCAATTGGTCAAAAAA-AAAAACTTTCATCACCAAAACTCACCGTTTTTGTATCCA 567

SPL_allele_2 ACAAGCAATTGATCAAAAAA-AAAAACTTTCATCACCAAAACTCACCGTTTTTGTATCCA 505

SPH_allele_1 ACAAGTAATTGGTCAAAAAA-AAAAACTTTCATCACCAAAACTCACCGTTTTTGTATCCA 565

TBR_allele_2 ACAAGTAATTGGTCAAAAAA-AAAAACTTTCATCACCAAAACTCACCGTTTTTGTATCCA 565

SPL_allele_1 ACAAGTAATTGGTCAAAAAA-AAAAACTTTCATCACCAAAACTCACCGTTTTTGTATCCA 565

TBR_allele_1 ACAAGCAATTGGTCAAAAAA-AAAAACTTTCATCACCAAAACTCACCGTTTTTGTATCCA 566

* *** **** * *** *** * ** ** ** ************** ******

PNT_allele_1 ATCTACGGTTACCTAGAAACTTCGAGAGACTCATTTTAACTATTTATACTCTTCTCATTT 658

JAM_allele_2 ATCTACGGTTGCCTAGAAACTTCGAGAGACTCATTTTAACTATTTATACTCTACTCATTT 640

JAM_allele_1 ATCTACGGTTGCCTAGAAACTTCGAGAGACTCATTTTAACTATTTATACTCTACTCATTT 638

VER_allele_1 ATCTACGGTTACCTAGAAACTTCGAGAGACTCATTTCAACTATTTATACTCTCCTCATTT 628

CRCQUM_allele_2 ATCTACGGTTACCCAGAAACTTCGAGAGACTCATTTCAACTATTTATACTCTTCTCATTT 621

IFO ATCTACGGTTACCTAGAAACTTCGAGAGACTCATTTCAACTATTTATACTCTTCTCATTT 627

Blv ATCTACGGTTACCTAGAAACTTCGAGAGACTCATTTCAACTATTTATACTCTTCTCATTT 620

PNT_allele_2 ATCTACGGTTACCTAGAAACCTCGAGAGACTCATTTCAACTATTTATACTCTTCTCATTT 624

LPH ATCTACGGTTACCTAGAAACTTCGAGAGACTCATTTCAACTATTTATACTCTTCTCATTT 624

VER_allele_2 ATCTACGGTTAGCTAGAAACTTCGAGAGACTCATTTCAACTATTTATACTCTTCTCATTT 625

AGF ATCTACGGTTACCTAGAAACTTCGAGAGACTCATTTCAACTATTTATACTCTTCTCATTT 599

Vio_allele_1 ATCTACGGTTACCTAGAAACTTCGAGAGACTCATTTCAACTATTTATACTCTTCTCATTT 599

CRCQUM_allele_1 ATCTACGGTTACCTAGAAACTTCGAGAGACTCATTTCAACTATTTATACTCTTCTCATTT 615

Vio_allele_2 ATCTACGGTTACCTAGAAACTTCGAGAGACTCATTTCAACTATTTATACTCTTCTCATTT 602

SPH_allele_2 ATCTACGGTTACCTAGAAACTTCGAGAGACTCATTTCAACTATTTATACTCTTCTCATTT 563

RAP_allele_2 ATCTACGGTTACCTAGAAACTTCGAGAGACTCATTTCAACTATTTATACTCTTCTCATTT 628

RAP_allele_1 ATCTACGGTTACCTAGAAACTTCGAGAGACTCATTTCAACTATTTATACTCTTCTCATTT 627

SPL_allele_2 ATCTACGGTTACCTAGAAACTTCGAGAGACTCATTTCAACTATTTATACTCTTTCTCATT 565

SPH_allele_1 ATCTACGGTTACCTAGAAACTTCGAGAGACTCATTTCAACTATTTATACTCTTTCTCATT 625

TBR_allele_2 ATCTACGGTTACCTAGAAACTTCGAGAGACTCATTTCAACTATTTATACTCTTTCTCATT 625

SPL_allele_1 ATCTACGGTTACCTAGAAACTTCGAGAGACTCATTTCAACTATTTATACTCTTTCTCATT 625

TBR_allele_1 ATCTACGGTTACCTAGAAACTTCGAGAGACTCATTTCAACTATTTATACTCTTTCTCATT 626

********** * ****** *************** *************** **

PNT_allele_1 TTTCCAATTTCATTCACATTATTATACCAAAAACAATCTGTAATTCCGTGATACTTTGCT 718

JAM_allele_2 TTTCTAGTTTCATTCACATA---ATACCAAAAACAATCTGTAATTCTGTGATACTTTGCT 697

JAM_allele_1 TTTCTAGTTTCATTCACATA---ATACCAAAAACAATCTGTAATTCTGTGATACTTTGCT 695

VER_allele_1 TTTTCCATGCTCATT--CACATTATACCAAAAACAATCTGTAATTCTGTGATACTTTGCT 686

CRCQUM_allele_2 TT-TCCATTTTCATT--CACATTTTACCAAAAACAATCTGTAATTCTGTGATACTTTGCT 678

IFO TT-TCCATTTTCATT--CACATTATACCAAAAACGATCTGTAATTCTGTGATACTTTGCT 684

Blv TT-TCCATTTTCATT—CACAT-----CCAAAAACGATCTGTAATTCTGTGATACTTTGCT 677

PNT_allele_2 TT-TCCATTTTCATT--CACATTATACCAAAAACGATCTGTAATTCTGTGATACTTTGCT 681

LPH TT-TCCATTTTCATT--CACATTATACCAAAAACGATCTGTAATTCTGTGATACTTTGCT 681

VER_allele_2 TT-TCCATTTTCATT--CACATTATACCAAAAACGATCTGTAATTCTGTGAGACTTTGCT 682

AGF TT-TCCATTTTCATT--AACATTATACCAAAAACGATCTGTAATTCTGTGATACTTTGCT 656

Vio_allele_1 TT-TCCATTTTCATT--AACATTATACCAAAAACGATCTGTAATTCTGTGATACTTTGCT 656

CRCQUM_allele_1 TT-TCCATTTTCATT--CACATTACACCAAAAACAATCTGTAATTCTGTGATACTTTGCT 672

Vio_allele_2 TT-TCCATTTTCATT--CACATTATACCAAAAACGATCTGTAATTCTGTGATACTTTGCT 659

SPH_allele_2 TT-TCCATTTTCATT--CACATTATACCAAAAACGATCTGTAATTCTGTGATACTTTGCT 620

RAP_allele_2 TT-TCCATTTTCATT--CATATTATACCAAAAACGATCTGTAATTCTGTGATACTTTGCT 685

RAP_allele_1 TT-TCCATTTTCATT--CATATTATACCAAAAACGATCTGTAATTCTGTGATACTTTGCT 684

SPL_allele_2 TT-TTCCATTTTCAT--TCACATTATACAAAAACAATCTGTAATTCTGTGATACTTTGCT 622

SPH_allele_1 TT-TTCCATTTTCAT--TCACATTATACAAAAACAATCTGTAATTCTGTGATACTTTGCT 682

TBR_allele_2 TT-TTCCATTTTCAT--TCACATTATACAAAAACAATCTGTAATTCTGTGATACTTTGCT 682

SPL_allele_1 TT-TTCCATTTTCAT--TCACATTATACAAAAACAATCTGTAATTCTGTGATACTTTGCT 682

TBR_allele_1 TT-TTCCATTTTCAT--TCACATTATACAAAAACAATCTGTAATTCTGTGATACTTTGCT 683

** ******* *********** **** ********

PNT_allele_1 TCACTAGTTAAAATTCATCTCCGTTTTATTCTTT----TTTGTTGTTGTGAAAAAGTTTG 774

JAM_allele_2 TTACTAGTTAAAATTCATCTCCGTTTTCTTCTTT----TTTGTTGTTGTGAAAAAGTTTG 753

JAM_allele_1 TTACTAGTTAAAATTCATCTCCGTTTTCTTCTTT----TTTGTTGTTGTGAAAAAGTTTG 751

VER_allele_1 TCACTAGTTGAAA----------------------------------------------- 699

CRCQUM_allele_2 TTACTAGTTGAAATTACTCTCTGTTT------TCT----TCGTTGTTGTGAAAAAGTTTG 728

IFO CTAGTAGTTAAAAAA-AAATTACTCTCTGTTTTCTTCTTTTGTTGTTGTGAAAAAGTTTG 743

Blv TTAGTATAAAAAAAAAAAATTACTCTCTGTTTTCTTCTTTTATTGTTGTGAAAAAGTTTG 737

PNT_allele_2 TTAGTAGTTAAAAAAAAAATTACTCTCTGTTTTCTTCTTTTATTGTTGTGAAAAAGTTTG 741

LPH TTAGTAGTTAAAAAA-AAATTACTCTCTGTTTTCTTCTTTTATTGTTGTGAAAAAGTTTG 740

VER_allele_2 TTAGTAGTTAAAAAATTACT------CTGTTTTCTTCTTTTATTGTTGTGAAAAAGTTTG 736

AGF TTAGTAGTTAAAAAATTACT------CTGTTTTCTTCTTATATTGTTGTGAAAAAGTTTG 710

Vio_allele_1 TTAGTAGTTAAAAAATTACT------CTGTTTTCTTCTTATATTGTTGTGAAAAAGTTTG 710

CRCQUM_allele_1 TTACTAGTTGAAATTACTCTCTGTTTTCTTC----------GTTGTTGTGAAAAAGTTTG 722

Vio_allele_2 TTACTAGTTGAAATTACTCTCTGTTTTCTTT----------ATTGTTGTGAAAAAGTTTG 709

SPH_allele_2 TTACTAGTTGAAATTACTCTCTGTTTTCTTC----------GTTGTTGTGAAAAAGTTTG 670

RAP_allele_2 TTACTAATTGAAATTACTCTCTGTTTTCTTC----------GTTGTTGTGAAAAAGTTTG 735

RAP_allele_1 TTACTAATTGAAATTACTCTCTGTTTTCTTC----------GTTGTTGTGAAAAAGTTTG 734

SPL_allele_2 TTACTAGTTGAAATTACTCTCTGTTTTCTTC----------ATTGTTGTGAAAAAGTTTG 672

SPH_allele_1 TTACTAGTTGAAATTACTCTCTGTTTTCTTC----------GTTGTTGTGAAAAAGTTTG 732

TBR_allele_2 TTACTAGTTGAAATTACTCTCTGTTTTCTTC----------GTTGTTGTGAAAAAGTTTG 732

SPL_allele_1 TTACTAGTTGAAATTACTCTCTGTTTTCTTC----------GTTGTTGTGAAAAAGTTTG 732

TBR_allele_1 TTACTAGTTGAAATTACTCTCTGTTTTCTTC----------GTTGTTGTGAAAAAGTTTG 733

* ** ***

PNT_allele_1 AAA 777

JAM_allele_2 AAA 756

JAM_allele_1 AAA 754

VER_allele_1 --- 699

CRCQUM_allele_2 AAA 731

IFO AAA 746

Blv AAA 740

PNT_allele_2 AAA 744

LPH AAA 743

VER_allele_2 AAA 739

AGF AAA 713

Vio_allele_1 AAA 713

CRCQUM_allele_1 AAA 725

Vio_allele_2 AAA 712

SPH_allele_2 AAA 673

RAP_allele_2 AAA 738

RAP_allele_1 AAA 737

SPL_allele_2 AAA 675

SPH_allele_1 AAA 735

TBR_allele_2 AAA 735

SPL_allele_1 AAA 735

TBR_allele_1 AAA 736
