# Supplementary material for: Allelic variants of a potato HEAT SHOCK COGNATE 70 gene confer improved tuber yield under a wide range of environmental conditions
Source: Food Energy Secur. 2022 Mar 15;12(1):e377. doi: 10.1002/fes3.377 (PMC10078605; doi:10.1002/fes3.377)
Supplement: Supplementary file 8 — Fig S8 [file FES3-12-0-s001.docx]

**Figure SX:** Clustal alignment of deduced amino acid sequences of all Hsc70 alleles in 13 wild potato accessions.

BLV_allele MAGKGEGPAIGIDLGTTYSCVGVWQHDRVEIIANDQGNRTTPSYVAFTDT**X**RLIGDAAKN 60

JAM_allele MAGKGEGPAIGIDLGTTYSCVGVWQHDRVEIIANDQGNRTTPSYVAFTDTERLIGDAAKN 60

PNT_allele MAGKGEGPAIGIDLGTTYSCVGVWQHDRVEIIANDQGNRTTPSYVAFTDTERLIGDAAKN 60

AGF_allele2 MAGKGEGPAIGIDLGTTYSCVGVWQHDRVEIIANDQGNRTTPSYVAFTDTERLIGDAAKN 60

SPH_allele MAGKGEGPAIGIDLGTTYSCVGVWQHDRVEIIANDQGNRTTPSYVAFTDTERLIGDAAKN 60

CRC_allele1 MAGKGEGPAIGIDLGTTYSCVGVWQHDRVEIIANDQGNRTTPSYVAFTDTERLIGDAAKN 60

CRC_allele2 MAGKGEGPAIGIDLGTTYSCVGVWQHDRVEIIANDQGNRTTPSYVAFTDTERLIGDAAKN 60

IFO_allele1 MAGKGEGPAIGIDLGTTYSCVGVWQHDRVEIIANDQGNRTTPSYVAFTDTERLIGDAAKN 60

IFO_allele2 MAGKGEGPAIGIDLGTTYSCVGVWQHDRVEIIANDQGNRTTPSYVAFTDTERLIGDAAKN 60

AGF_allele1 MAGKGEGPAIGIDLGTTYSCVGVWQHDRVEIIANDQGNRTTPSYVAFTDTERLIGDAAKN 60

VIO_allele1 MAGKGEGPAIGIDLGTTYSCVGVWQHDRVEIIANDQGNRTTPSYVAFTDTERLIGDAAKN 60

VIO_allele2 MAGKGEGPAIGIDLGTTYSCVGVWQHDRVEIIANDQGNRTTPSYVAFTDTERLIGDAAKN 60

SPL_allele1 MAGKGEGPAIGIDLGTTYSCVGVWQHDRVEIIANDQGNRTTPSYVAFTDTERLIGDAAKN 60

RAP_allele2 MAGKGEGPAIGIDLGTTYSCVGVWQHDRVEIIANDQGNRTTPSYVAFTDTERLIGDAAKN 60

VER_allele2 MAGKGEGPAIGIDLGTTYSCVGVWQHDRVEIIANDQGNRTTPSYVAFTDTERLIGDAAKN 60

LPH_allele MAGKGEGPAIGIDLGTTYSCVGVWQHDRVEIIANDQGNRTTPSYVAFTDTERLIGDAAKN 60

RAP_allele1 MAGKGEGPAIGIDLGTTYSCVGVWQHDRVEIIANDQGNRTTPSYVAFTDTERLIGDAAKN 60

VER_allele1 MAGKGEGPAIGIDLGTTYSCVGVWQHDRVEIIANDQGNRTTPSYVAFTDTERLIGDAAKN 60

TBR_allele MAGKGEGPAIGIDLGTTYSCVGVWQHDRVEIIANDQGNRTTPSYVAFTDTERLIGDAAKN 60

SPL_allele2 MAGKGEGPAIGIDLGTTYSCVGVWQHDRVEIIANDQGNRTTPSYVAFTDTERLIGDAAKN 60

************************************************** *********

BLV_allele QVAMNPTNTVFDAKRLIGRRFSDPSVQSDMKLWPFKVIPGPADKPMIVVNYKGEEKEFSA 120

JAM_allele QVAMNPTNTVFDAKRLIGRRFSDPSVQSDMKLWPFKVIPGPADKPMIVVNYKGEEKEFSA 120

PNT_allele QVAMNPTNTVFDAKRLIGRRFSDPSVQSDMKLWPFKVIPGPADKPMIVVNYKGEEKEFSA 120

AGF_allele2 QVAMNPTNTVFDAKRLIGRRF**T**DPSVQSDMKLWPFKVIPGPADKPMIVVNYKGEEKEFSA 120

SPH_allele QVAMNPTNTVFDAKRLIGRRFSDPSVQSDMKLWPFKVIPGPADKPMIVVNYKGEEKEFSA 120

CRC_allele1 QVAMNPTNTVFDAKRLIGRRFSDPSVQSDMKLWPFKVIPGPADKPMIVVNYKGEEKEFSA 120

CRC_allele2 QVAMNPTNTVFDAKRLIGRRFSDPSVQSDMKLWPFKVIPGPADKPMIVVNYKGEEKEFSA 120

IFO_allele1 QVAMNPTNTVFDAKRLIGRRFSDPSVQSDMKLWPFKVIPGPADKPMIVVNYKGEEKEFSA 120

IFO_allele2 QVAMNPTNTVFDAKRLIGRRFSDPSVQSDMKLWPFKVIPGPADKPMIVVNYKGEEKEFSA 120

AGF_allele1 QVAMNPTNTVFDAKRLIGRRF**T**DPSVQSDMKLWPFKVIPGPADKPMIVVNYKGEEKEFSA 120

VIO_allele1 QVA**T**NPTNTVFDAKRLIGRRFSDPSVQSDMKLWPFKVIPGPADKPMIVVNYKGEEKEFSA 120

VIO_allele2 QVA**T**NPTNTVFDAKRLIGRRFSDPSVQSDMKLWPFKVIPGPADKPMIVVNYKGEEKEFSA 120

SPL_allele1 QVAMNPTNTVFDAKRLIGRRFSDPSVQSDMKLWPFKVIPGPADKPMIVVNYKGEEKEFSA 120

RAP_allele2 QVAMNPTNTVFDAKRLIGRRFSDPSVQSDMKLWPFKVIPGPADKPMIVVNYKGEEKEFSA 120

VER_allele2 QVAMNPTNTVFDAKRLIGRRFSDPSVQSDMKLWPFKVIPGPADKPMIVVNYKGEEKEFSA 120

LPH_allele QVAMNPTNTVFDAKRLIGRRFSDPSVQSDMKLWPFKVIPGPADKPMIVVNYKGEEKEFSA 120

RAP_allele1 QVAMNPTNTVFDAKRLIGRRFSDPSVQSDMKLWPFKVIPGPADKPMIVVNYKGEEKEFSA 120

VER_allele1 QVAMNPTNTVFDAKRLIGRRFSDPSVQSDMKLWPFKVIPGPADKPMIVVNYKGEEKEFSA 120

TBR_allele QVAMNPTNTVFDAKRLIGRRFSDPSVQSDMKLWPFKVIPGPADKPMIVVNYKGEEKEFSA 120

SPL_allele2 QVAMNPTNTVFDAKRLIGRRFSDPSVQSDMKLWPFKVIPGPADKPMIVVNYKGEEKEFSA 120

*** *****************:**************************************

BLV_allele EEISSMVLIKMKEIAEAFLGITIKNAVVTVPAYFNDSQRQATKDAGTISGLNVMRIINEP 180

JAM_allele EEISSMVLIKMKEIAEAFLGITIKNAVVTVPAYFNDSQRQATKDAGTISGLNVMRIINEP 180

PNT_allele EEISSMVLIKMKEIAEAFLGITIKNAVVTVPAYFNDSQRQATKDAGTISGLNVMRIINEP 180

AGF_allele2 EEISSMVLIKMKEIAEAFLGITIKNAVVTVPAYFNDSQRQATKDAGTISGLNVMRIINEP 180

SPH_allele EEISSMVLIKMKEIAEAFLGITIKNAVVTVPAYFNDSQRQATKDAGTISGLNVMRIINEP 180

CRC_allele1 EEISSMVLIKMKEIAEAFLGITIKNAVVTVPAYFNDSQRQATKDAGTISGLNVMRIINEP 180

CRC_allele2 EEISSMVLIKMKEIAEAFLGITIKNAVVTVPAYFNDSQRQATKDAGTISGLNVMRIINEP 180

IFO_allele1 EEISSMVLIKMKEIAEAFLGITIKNAVVTVPAYFNDSQRQATKDAGTISGLNVMRIINEP 180

IFO_allele2 EEISSMVLIKMKEIAEAFLGITIKNAVVTVPAYFNDSQRQATKDAGTISGLNVMRIINEP 180

AGF_allele1 EEISSMVLIKMKEIAEAFLGITIKNAVVTVPAYFNDSQRQATKDAGTISGLNVMRIINEP 180

VIO_allele1 EEISSMVLIKMKEIAEAFLGITIKNAVVTVPAYFNDSQRQATKDAGTISGLNVMRIINEP 180

VIO_allele2 EEISSMVLIKMKEIAEAFLGITIKNAVVTVPAYFNDSQRQATKDAGTISGLNVMRIINEP 180

SPL_allele1 EEISSMVLIKMKEIAEAFLGITI**N**NAVVTVPAYFNDSQRQATKDAGTISGLNVMRIINEP 180

RAP_allele2 EEISSMVLIKMKEIAEAFLGITIKNAVVTVPAYFNDSQRQATKDAGTISGLNVMRIINEP 180

VER_allele2 EEISSMVLIKMKEIAEAFLGITIKNAVVTVPAYFNDSQRQATKDAGTISGLNVMRIINEP 180

LPH_allele EEISSMVLIKMKEIAEAFLGITIKNAVVTVPAYFNDSQRQATKDAGTISGLNVMRIINEP 180

RAP_allele1 EEISSMVLIKMKEIAEAFLGITIKNAVVTVPAYFNDSQRQATKDAGTISGLNVMRIINEP 180

VER_allele1 EEISSMVLIKMKEIAEAFLGITIKNAVVTVPAYFNDSQRQATKDAGTISGLNVMRIINEP 180

TBR_allele EEISSMVLIKMKEIAEAFLGITIKNAVVTVPAYFNDSQRQATKDAGTISGLNVMRIINEP 180

SPL_allele2 EEISSMVLIKMKEIAEAFLGITIKNAVVTVPAYFNDSQRQATKDAGTISGLNVMRIINEP 180

***********************:************************************

BLV_allele TAAAIAYGLDKKSSSTGEKTVLIFDLGGGTFDVSLLTIEEGIFEVKATAGDTHLGGEDFD 240

JAM_allele TAAAIAYGLDKKSSSTGEKTVLIFDLGGGTFDVSLLTIEEGIFEVKATAGDTHLGGEDFD 240

PNT_allele TAAAIAYGLDKKSSSTGEKTVLIFDLGGGTFDVSLLTIEEGIFEVKATAGDTHLGGEDFD 240

AGF_allele2 TAAAIAYGLDKKSSSTGEKTVLIFDLGGGTFDVSLLTIEEGIFEVKATAGDTHLGGEDFD 240

SPH_allele TAAAIAYGLDKKSSSTGEKTVLIFDLGGGTFDVSLLTIEEGIFEVKATAGDTHLGGEDFD 240

CRC_allele1 TAAAIAYGLDKKSSSTGEKTVLIFDLGGGTFDVSLLTIEEGIFEVKATAGDTHLGGEDFD 240

CRC_allele2 TAAAIAYGLDKKSSSTGEKTVLIFDLGGGTFDVSLLTIEEGIFEVKATAGDTHLGGEDFD 240

IFO_allele1 TAAAIAYGLDKKSSSTGEKTVLIFDLGGGTFDVSLLTIEEGIFEVKATAGDTHLGGEDFD 240

IFO_allele2 TAAAIAYGLDKKSSSTGEKTVLIFDLGGGTFDVSLLTIEEGIFEVKATAGDTHLGGEDFD 240

AGF_allele1 TAAAIAYGLDKKSSSTGEKTVLIFDLGGGTFDVSLLTIEEGIFEVKATAGDTHLGGEDFD 240

VIO_allele1 TAAAIAYGLDKKSSSTGEKTVLIFDLGGGTFDVSLLTIEEGIFEVKATAGDTHLGGEDFD 240

VIO_allele2 TAAAIAYGLDKKSSSTGEKTVLIFDLGGGTFDVSLLTIEEGIFEVKATAGDTHLGGEDFD 240

SPL_allele1 TAAAIAYGLDKKSSSTGEKTVLIFDLGGGTFDVSLLTIEEGIFEVKATAGDTHLGGEDFD 240

RAP_allele2 TAAAIAYGLDKKSSSTGEKTVLIFDLGGGTFDVSLLTIEEGIFEVKATAGDTHLGGEDFD 240

VER_allele2 TAAAIAYGLDKKSSSTGEKTVLIFDLGGGTFDVSLLTIEEGIFEVKATAGDTHLGGEDFD 240

LPH_allele TAAAIAYGLDKKSSSTGEKTVLIFDLGGGTFDVSLLTIEEGIFEVKATAGDTHLGGEDFD 240

RAP_allele1 TAAAIAYGLDKKSSSTGEKTVLIFDLGGGTFDVSLLTIEEGIFEVKATAGDTHLGGEDFD 240

VER_allele1 TAAAIAYGLDKKSSSTGEKTVLIFDLGGGTFDVSLLTIEEGIFEVKATAGDTHLGGEDFD 240

TBR_allele TAAAIAYGLDKKSSSTGEKTVLIFDLGGGTFDVSLLTIEEGIFEVKATAGDTHLGGEDFD 240

SPL_allele2 TAAAIAYGLDKKSSSTGEKTVLIFDLGGGTFDVSLLTIEEGIFEVKATAGDTHLGGEDFD 240

************************************************************

BLV_allele NRMVNHFVQEFKRKHKKDISGNPRALRRLRTACERAKRTLSSTAQTTIEIDSLYEGIDFY 300

JAM_allele NRMVNHFVQEFKRKHKKDISGNPRALRRLRTACERAKRTLSSTAQTTIEIDSLYEGIDFY 300

PNT_allele NRMVNHFVQEFKRKHKKDISGNPRALRRLRTACERAKRTLSSTAQTTIEIDSLYEGIDFY 300

AGF_allele2 NRMVNHFVQEFKRKHKKDISGNPRALRRLRTACERAKRTLSSTAQTTIEIDSLYEGIDFY 300

SPH_allele NRMVNHFVQEFKRKHKKDISGNPRALRRLRTACERAKRTLSSTAQTTIEIDSLYEGIDFY 300

CRC_allele1 NRMVNHFVQEFKRKHKKDISGNPRALRRLRTACERAKRTLSSTAQTTIEIDSLYEGIDFY 300

CRC_allele2 NRMVNHFVQEFKRKHKKDISGNPRALRRLRTACERAKRTLSSTAQTTIEIDSLYEGIDFY 300

IFO_allele1 NRMVNHFVQEFKRKHKKDISGNPRALRRLRTACERAKRTLSSTAQTTIEIDSLYEGIDFY 300

IFO_allele2 NRMVNHFVQEFKRKHKKDISGNPRALRRLRTACERAKRTLSSTAQTTIEIDSLYEGIDFY 300

AGF_allele1 NRMVNHFVQEFKRKHKKDISGNPRALRRLRTACERAKRTLSSTAQTTIEIDSLYEGIDFY 300

VIO_allele1 NRMVNHFVQEFKRKHKKDISGNPRALRRLRTACERAKRTLSSTAQTTIEIDSLYEGIDFY 300

VIO_allele2 NRMVNHFVQEFKRKHKKDISGNPRALRRLRTACERAKRTLSSTAQTTIEIDSLYEGIDFY 300

SPL_allele1 NRMVNHFVQEFKRKHKKDISGNPRALRRLRTACERAKRTLSSTAQTTIEIDSLYEGIDFY 300

RAP_allele2 NRMVNHFVQEFKRKHKKDISGNPRALRRLRTACERAKRTLSSTAQTTIEIDSLYEGIDFY 300

VER_allele2 NRMVNHFVQEFKRKHKKDISGNPRALRRLRTACERAKRTLSSTAQTTIEIDSLYEGIDFY 300

LPH_allele NRMVNHFVQEFKRKHKKDISGNPRALRRLRTACERAKRTLSSTAQTTIEIDSLYEGIDFY 300

RAP_allele1 NRMVNHFVQEFKRKHKKDISGNPRALRRLRTACERAKRTLSSTAQTTIEIDSLYEGIDFY 300

VER_allele1 NRMVNHFVQEFKRKHKKDISGNPRALRRLRTACERAKRTLSSTAQTTIEIDSLYEGIDFY 300

TBR_allele NRMVNHFVQEFKRKHKKDISGNPRALRRLRTACERAKRTLSSTAQTTIEIDSLYEGIDFY 300

SPL_allele2 NRMVNHFVQEFKRKHKKDISGNPRALRRLRTACERAKRTLSSTAQTTIEIDSLYEGIDFY 300

************************************************************

BLV_allele TTITRARFEELNMDLFRKCMEPVEKCLRDAKID**R**SGVHDIVLVGGSTRIPKVQQLLQDFF 360

JAM_allele TTITRARFEELNMDLFRKCMEPVEKCLRDAKIDKSGVHDIVLVGGSTRIPKVQQLLQDFF 360

PNT_allele TTITRARFEELNMDLFRKCMEPVEKCLRDAKIDKSGVHDIVLVGGSTRIPKVQQLLQDFF 360

AGF_allele2 TTITRARFEELNMDLFRKCMEPVEKCLRDAKIDKSGVHDIVLVGGSTRIPKVQQLLQDFF 360

SPH_allele TTITRARFEELNMDLFRKCMEPVEKCLRDAKIDKSGVHDIVLVGGSTRIPKVQQLLQDFF 360

CRC_allele1 TTITRARFEELNMDLFRKCMEPVEKC**F**RDAKIDKSGVHDIVLVGGSTRIPKVQQLLQDFF 360

CRC_allele2 TTITRARFEELNMDLFRKCMEPVEKC**F**RDAKIDKSGVHDIVLVGGSTRIPKVQQLLQDFF 360

IFO_allele1 TTITRARFEELNMDLFRKCMEPVEKCLRDAKIDKSGVHDIVLVGGSTRIPKVQQLLQDFF 360

IFO_allele2 TTITRARFEELNMDLFRKCMEPVEKCLRDAKIDKSGVHDIVLVGGSTRIPKVQQLLQDFF 360

AGF_allele1 TTITRARFEELNMD**F**FRKCMEPVEKCLRDAKIDKSGVHDIVLVGGSTRIPKVQQLLQDFF 360

VIO_allele1 TTITRARFEELNMDLFRKCMEPVEKCLRDAKIDKSGVHDIVLVGGSTRIPKVQQLLQDFF 360

VIO_allele2 TTITRARFEELNMDLFRKCMEPVEKCLRDAKIDKSGVHDIVLVGGSTRIPKVQQLLQDFF 360

SPL_allele1 TTITRARFEELNMDLFRKCMEPVEKCLRDAKIDKSGVHDIVLVGGSTRIPKVQQLLQDFF 360

RAP_allele2 TTITRARFEELNMDLFRKCMEPVEKCLRDAKIDKSGVHDIVLVGGSTRIPKVQQLLQDFF 360

VER_allele2 TTITRARFEELNMDLFRKCMEPVEKCLRDAKIDKSGVHDIVLVGGSTRIPKVQQLLQDFF 360

LPH_allele TTITRARFEELNMDLFRKCMEPVEKCL**X**DAKIDKSGVHDIVLVGGSTRIPKVQQLLQDFF 360

RAP_allele1 TTITRARFEELNMDLFRKCMEPVEKCLRDAKIDKSGVHDIVLVGGSTRIPKVQQLLQDFF 360

VER_allele1 TTITRARFEELNMDLFRKCMEPVEKCLRDAKIDKSGVHDIVLVGGSTRIPKVQQLLQDFF 360

TBR_allele TTITRARFEELNMDLFRKCMEPVEKCLRDAKIDKSGVHDIVLVGGSTRIPKVQQLLQDFF 360

SPL_allele2 TTITRARFEELNMDLFRKCMEPVEKCLRDAKIDKSGVHDIVLVGGSTRIPKVQQLLQDFF 360

**************:***********: *****:**************************

BLV_allele NGKELCKSINPDEAVAYGAAVQAAILSGEGNEKVQDLLLLDVTPLSLGLETAGGVMTTLI 420

JAM_allele NGKELCKSINPDEAVAYGAAVQAAILSGEGNEKVQDLLLLDVTPLSLGLETAGGVMTTLI 420

PNT_allele NGKELCKSINPDEAVAYGAAVQAAILSGEGNEKVQDLLLLDVTPLSLGLETAGGVMTTLI 420

AGF_allele2 NGKELCKSINPDEAVAYGAAVQAAILSGEGNEKVQDLLLLDVTPLSLGLETAGGVMTTLI 420

SPH_allele NGKELCKSINPDEAVAYGAAVQAAILSGEGNEKVQDLLLLDVTPLSLGLETAGGVMTTLI 420

CRC_allele1 NGKELCKSINPDEAVAYGAAVQAAILSGEGNEKVQDLLLLDVTPLSLGLETAGGVMTTLI 420

CRC_allele2 NGKELCKSINPDEAVAYGAAVQAAILSGEGNEKVQDLLLLDVTPLSLGLETAGGVMTTLI 420

IFO_allele1 NGKELCKSINPDEAVAYGAAVQAAILSGEGNEKVQDLLLLDVTPLSLGLETAGGVMTTLI 420

IFO_allele2 NGKELCKSINPDEAVAYGAAVQAAILSGEGNEKVQDLLLLDVTPLSLGLETAGGVMTTLI 420

AGF_allele1 NGKELCKSINPDEAVAYGAAVQAAILSGEGNEKVQDLLLLDVTPLSLGLETAGGVMTTLI 420

VIO_allele1 NGKELCKSINPDEAVAYGAAVQAAILSGEGNEKVQDLLLLDVTPLSLGLETAGGVMTTLI 420

VIO_allele2 NGKELCKSINPDEAVAYGAAVQAAILSGEGNEKVQDLLLLDVTPLSLGLETAGGVMTTLI 420

SPL_allele1 NGKELCKSINPDEAVAYGAAVQAAILSGEGNEKVQDLLLLDVTPLSLGLETAGGVMTTLI 420

RAP_allele2 NGKELCKSINPDEAVAYGAAVQAAILSGEGNEKVQDLLLLDVTPLSLGLETAGGVMTTLI 420

VER_allele2 NGKELCKSINPDEAVAYGAAVQAAILSGEGNEKVQDLLLLDVTPLSLGLETAGGVMTTLI 420

LPH_allele NGKELCKSINPDEAVAYGAAVQAAILSGEGNEKVQDLLLLDVTPLSLGLETAGGVMTTLI 420

RAP_allele1 NGKELCKSINPDEAVAYGAAVQAAILSGEGNEKVQDLLLLDVTPLSLGLETAGGVMTTLI 420

VER_allele1 NGKELCKSINPDEAVAYGAAVQAAILSGEGNEKVQDLLLLDVTPLSLGLETAGGVMTTLI 420

TBR_allele NGKELCKSINPDEAVAYGAAVQAAILSGEGNEKVQDLLLLDVTPLSLGLETAGGVMTTLI 420

SPL_allele2 NGKELCKSINPDEAVAYGAAVQAAILSGEGNEKVQDLLLLDVTPLSLGLETAGGVMTTLI 420

************************************************************

BLV_allele PRNTTIPTKKEQVFSTYSDNQPGV**S**IQVYEGERARTRDNNLLGKFELTGIPPAPRGVPQI 480

JAM_allele PRNTTIPTKKEQVFSTYSDNQPGVLIQVYEGERARTRDNNLLGKFELTGIPPAPRGVPQI 480

PNT_allele PRNTTIPTKKEQVFSTYSDNQPGVLIQVYEGERARTRDNNLLGKFELTGIPPAPRGVPQI 480

AGF_allele2 PRNTTIPTKKEQVFSTYSDNQPGVLIQVYEGERARTRDNNLLGKFELTGIPPAPRGVPQI 480

SPH_allele PRNTTIPTKKEQVFSTYSDNQPGVLIQVYEGERARTRDNNLLGKFELTGIPPAPRGVPQI 480

CRC_allele1 PRNTTIPTKKEQVFSTYSDNQPGVLIQVYEGERARTRDNNLLGKFELTGIPPAPRGVPQI 480

CRC_allele2 PRNTTIPTKKEQVFSTYSDNQPGVLIQVYEGERARTRDNNLLGKFELTGIPPAPRGVPQI 480

IFO_allele1 PRNTTIPTKKEQVFSTYSDNQPGVLIQVYEGERARTRDNNLLGKFELTGIPPAPRGVPQI 480

IFO_allele2 PRNTTIPTKKEQVFSTYSDNQPGVLIQVYEGERARTRDNNLLGKFELTGIPPAPRGVPQI 480

AGF_allele1 PRNTTIPTKKEQVFSTYSDNQPGVLIQVYEGERARTRDNNLLGKFELTGIPPAPRGVPQI 480

VIO_allele1 PRNTTIPTKKEQVFSTYSDNQPGVLIQVYEGERARTRDNNLLGKFELTGIPPAPRGVPQI 480

VIO_allele2 PRNTTIPTKKEQVFSTYSDNQPGVLIQVYEGERARTRDNNLLGKFELTGIPPAPRGVPQI 480

SPL_allele1 PRNTTIPTKKEQVFSTYSDNQPGVLIQVYEGERARTRDN**T**LLGKFELTGIPPAPRGVPQI 480

RAP_allele2 PRNTTIPTKKEQVFSTYSDNQPGVLIQVYEGERARTRDNNLLGKFELTGIPPAPRGVPQI 480

VER_allele2 PRNTTIPTKKEQVFSTYSDNQPGVLIQVYEGERARTRDNNLLGKFELTGIPPAPRGVPQI 480

LPH_allele PRNTTIPTKKEQVFSTYSDNQPGVLIQVYEGERARTRDNNLLGKFELTGIPPAPRGVPQI 480

RAP_allele1 PRNTTIPTKKEQVFSTYSDNQPGVLIQVYEGERARTRDNNLLGKFELTGIPPAPRGVPQI 480

VER_allele1 PRNTTIPTKKEQVFSTYSDNQPGVLIQVYEGERARTRDNNLLGKFELTGIPPAPRGVPQI 480

TBR_allele PRNTTIPTKKEQVFSTYSDNQPGVLIQVYEGERARTRDNNLLGKFELTGIPPAPRGVPQI 480

SPL_allele2 PRNTTIPTKKEQVFSTYSDNQPGVLIQVYEGERARTRDNNLLGKFELTGIPPAPRGVPQI 480

************************ **************.********************

BLV_allele TVCFDIDANGILNV**P**AEDKTTGQKNKITITNDKGRLSKDEIEKMVQEAE**R**YKAEDEEHKK 540

JAM_allele TVCFDIDANGILNVSAEDKTTGQKNKITITNDKGRLSKDEIEKMVQEAEKYKAEDEEHKK 540

PNT_allele TVCFDIDANGILNVSAEDKTTGQKNKITITNDKGRLSKDEIEKMVQEAE**K**YKAEDEEHKK 540

AGF_allele2 TVCFDIDANGILNVSAEDKTTGQKNKITITNDKGRLSKDEIEKMVQEAERYKAEDEEHKK 540

SPH_allele TVCFDIDANGILNVSAEDKTTGQKNKITITNDKGRLSKDEIEKMVQEAERYKAEDEEHKK 540

CRC_allele1 TVCFDIDANGILNVSAEDKTTGQKNKITITNDKGRLSKDEIEKMVQEAERYKAEDEEHKK 540

CRC_allele2 TVCFDIDANGILNVSAEDKTTGQKNKITITNDKGRLSKDEIEKMVQEAERYKAEDEEHKK 540

IFO_allele1 TVCFDIDANGILNVSAEDKTTGQKNKITITNDKGRLSKDEIEKMVQEAERYKAEDEEH**R**K 540

IFO_allele2 TVCFDIDANGILNVSAEDKTTGQKNKITITNDKGRLSKDEIEKMVQEAERYKAEDEEH**R**K 540

AGF_allele1 TVCFDIDANGILNVSAEDKTTGQKNKITITNDKGRLSKDEIEKMVQEAERYKAEDEEHKK 540

VIO_allele1 TVCFDIDANGILNVSAEDKTTGQKNKITITNDKGRLSKDEIEKMVQEAERYKAEDEEHKK 540

VIO_allele2 TVCFDIDANGILNVSAEDKTTGQKNKITITNDKGRLSKDEIEKMVQEAERYKAEDEEHKK 540

SPL_allele1 TVCFDIDANGILNVSAEDKTTGQKNKITITNDKGRLSKDEIEKMVQEAERYKAEDEEHKK 540

RAP_allele2 TVCFDIDANGILNVSAEDKTTGQKNKITITNDKGRLSKDEIEKMVQEAERYKAEDEEHKK 540

VER_allele2 TVCFDIDANGILNVSAEDKTTGQKNKITITNDKGRLSKDEIEKMVQEAERYKAEDEEHKK 540

LPH_allele TVCFDIDANGILNVSAEDKTTGQKNKITITNDKGRLSKDEIEKMVQEAERYKAEDEEHKK 540

RAP_allele1 TVCFDIDANGILNVSAEDKTTGQKNKITITNDKGRLSKDEIEKMVQEAERYKAEDEEHKK 540

VER_allele1 TVCFDIDANGILNVSAEDKTTGQKNKITITNDKGRLSKDEIEKMVQEAERYKAEDEEHKK 540

TBR_allele TVCFDIDANGILNVSAEDKTTGQKNKITITNDKGRLSKDEIEKMVQEAERYKAEDEEHKK 540

SPL_allele2 TVCFDIDANGILNVSAEDKTTGQKNKITITNDKGRLSKDEIEKMVQEAERYKAEDE**G**HKK 540

************** **********************************:****** *:*

BLV_allele KVEAKNALENYAYNMRNTIKD**E**KIASKLSPEEKQKIEDSVEQAIQWLDGNQLAEADEFED 600

JAM_allele KVEAKNALENYAYNMRNTIKD**E**KIASKLSP**D**EKKKIEDSVEQAIQWLDGNQLAEADEFED 600

PNT_allele KVEAKNALENYAYNMRNTIKD**E**KIASKLSP**D**EKKKIEDSVEQAIQWLDGNQLAEADEFED 600

AGF_allele2 KVEAKNALENYAYNMRNTIKD**D**KIASKLSP**D**EKKKIEDSVEQAIQWLDGNQLAEADEFED 600

SPH_allele KVEAKNALENYAYNMRNTIKD**D**KIASKLSP**D**EKKKIEDSVEQAIQWLDGNQLAEADEFED 600

CRC_allele1 KVEAKNALENYAYNMRNTIKD**D**KIASKLSPEEKQKIEDSVEQAIQWLDGNQLAEADEFED 600

CRC_allele2 KVEAKNALENYAYNMRNTIKD**D**KIASKLSPEEKQKIEDSVEQAIQWLDGNQLAEADEFED 600

IFO_allele1 KVEAKNALENYAYNMRNTIKD**E**KIASKLSPEEKQKIEDSVEQAIQWLDGNQLAEADEFED 600

IFO_allele2 KVEAKNALENYAYNMRNTIKD**E**KIASKLSPEEKQKIEDSVEQAIQWLDGNQLAEADEFED 600

AGF_allele1 KVEAKNALENYAYNMRNTIKD**E**KIASKLSPEEKQKIEDSVEQAIQWLDGNQLAEADEFED 600

VIO_allele1 KVEAKNALENYAYNMRNTIKD**E**KIASKLSPEEKQKIEDSVEQAIQWLDGNQLAEADEFED 600

VIO_allele2 KVEAKNALENYAYNMRNTIKD**D**KIASKLSPEEKQKIEDSVEQAIQWLDGNQLAEADEFED 600

SPL_allele1 KVEAKNALENYAYNMRNTIKD**D**KIASKLSPEEKQKIEDSVEQAIQWLDGNQLAEADEFED 600

RAP_allele2 KVEAKNALENYAYNMRNTIKD**D**KIASKLSPEEKQKIEDSVEQAIQWLDGNQLAEADEFED 600

VER_allele2 KVEAKNALENYAYNMRNTIKD**D**KIASKLSPEEKQKIED**L**VEQAIQWLDGNQLAEADEFED 600

LPH_allele KVEAKNALENYAYNMRNTIKD**D**KIASKLSPEEKQKIEDSVEQAIQWLDGNQLAEADEFED 600

RAP_allele1 KVEAKNALENYAYNMRNTIKD**D**KIASKLSPEEKQKIEDSVEQAIQWLDGNQLAEADEFED 600

VER_allele1 KVEAKNALENYAYNMRNTIKD**D**KIASKLSPEEKQKIEDSVEQAIQWLDGNQLAEADEFED 600

TBR_allele KVEAKNALENYAYNMRNTIKD**D**KIASKLSPEEKQKIEDSVEQAIQWLDGNQLAEADEFED 600

SPL_allele2 KVEAKNALENYAYNMRNTIKD**D**KIASKLSPEEKQKIEDSVEQAIQWLDGNQLAEADEFED 600

*********************:********:**:**** *********************

BLV_allele KMKELESICNPIIAKMYQGGAGGPDMAGGMDEDGPSAGASGAGAGPKIEEVD 652

JAM_allele KMKELESICNPIIAKMYQGGAGGPDMAGGMDEDGPSAGASGAGAGPKIEEVD 652

PNT_allele KMKELESICNPIIAKMYQGGAGGPDMAGGMDEDGPSAGASGAGAGPKIEEVD 652

AGF_allele2 KMKELESICNPIIAKMYQGGAGGPDMAGGMDEDGPSAGASGAGAGPKIEEVD 652

SPH_allele KMKELESICNPIIAKMYQGGAGGPDMAGGMDEDGPSAGASGAGAGPKIEEVD 652

CRC_allele1 KMKELESICNPIIAKMYQGG**---**ADMAGGMDEDGPSAGASGAGAGPKIEEVD 649

CRC_allele2 KMKELESICNPIIAKMYQGG**---**ADMAGGMDEDGPSAGASGAGAGPKIEEVD 649

IFO_allele1 KMKELE**T**ICNPIIAKMYQGG**V**GGPDMAGGMDEDGPSAGASGAGAGPKIEEVD 652

IFO_allele2 KMKELE**T**ICNPIIAKMYQGGVGGPDMAGGMDEDGPSAGASGAGAGPKIEEVD 652

AGF_allele1 KMKELESICNPIIAKMYQGGAGGPDMAGGMDEDGPSAGASGAGAGPKIEEVD 652

VIO_allele1 KMKELESICNPIIAKMYQGGAGGPDMAGGMDEDGPSAGASGAGAGPKIEEVD 652

VIO_allele2 KMKELESICNPIIAKMYQGGAGGPDMAGGMDEDGPSAGASGAGAGPKIEEVD 652

SPL_allele1 KMKELESICNPIIAKMYQGGAGGPDMAGGMDEDGPSAGASGAGAGPKIEEVD 652

RAP_allele2 KMK**G**LESICNPIIAKMYQGGAGGPDMAGGMDEDGPSAGASG**T**GAGPKIEEVD 652

VER_allele2 KMKELESICNPIIAKMYQGGAGGPDMAGGMDEDGPSAGASGAGAGPKIEEVD 652

LPH_allele KMKELESICNPIIAKMYQGGAGGPDMAGGMDEDGPSAGASGAGAGPKIEEVD 652

RAP_allele1 KMKELESICNPIIAKMYQGGAGGPDMAGGMDEDGPSAGASGAGAGPKIEEVD 652

VER_allele1 KMKELESICNPIIAKMYQGGAGGPDMAGGMDEDGPSAGASGAGAGPKIEEVD 652

TBR_allele KMKELESICNPIIAKMYQGGAGGPDMAGGMDEDGPSAGASGAGAGPKIEEVD 652

SPL_allele2 KMKELESICNPIIAKMYQGGAGGPDMAGGMDEDGPSAGASGAGAGPKIEEVD 652

*** **:************* *****************:**********
