# Supplementary material for: Allelic variants of a potato HEAT SHOCK COGNATE 70 gene confer improved tuber yield under a wide range of environmental conditions
Source: Food Energy Secur. 2022 Mar 15;12(1):e377. doi: 10.1002/fes3.377 (PMC10078605; doi:10.1002/fes3.377)
Supplement: Supplementary file 11 — Supplementary Material [file FES3-12-0-s008.docx]

**Supplementary Figure S1:** Average FW tuber yield of the different HSc70 genotype classes from harvests at 40, 70 and 90 days after planting. Data from Sang’alo 2018 trial.

**Supplementary Figure S2:** Comparison of tuber FW yield between seasons for the Makoka trials (Figure S2a) and at the Sang’alo site (Figure S2b)

**Supplementary Figure S3:** Tuber yield score for 06H1 genotypes comparing UK trials from 2009 and 2010 for the 60 genotypes selected for the trials in Kenya and Malawi.

**Supplementary Figure 4: A)** Potato tuber yield GWAS Manhattan plots for all genetic effect (a, additive; b, general; c, 1-dom; d, 2-dom; e, diplo-additive; f, diplo-general) and GWAS models employed in the study. Significance threshold (dashed line) is based on the genome-wide False Discovery Rate (FDR) correction (α = 0.10) method. **(B)** Q-Q plots comparing the inflation of p-values for the four principal GWAS models employed for tuber yield under all genetic effect models. Red circles: Naïve model; Green squares: K model; Blue diamonds: Q model; and Black triangles: QK model. Red line indicates p-values under the expected normal distribution. Numerical value against each GWAS model name represents genomic control inflation factor (λGC) for that model.

**Supplementary Figure S5:** Relative cell membrane injury after exposure to 40^o^C heat stress for 24 h in leaves from CPC accessions compared with control plants kept at 20^o^C as assessed by an electrolyte leakage assay. Data are presented as mean ± SE, n = 6.

**Supplementary Figure S6:** CLUSTAL O(1.2.4) multiple sequence alignment of CPC promoter sequence. The TA repeat region is highlighted in red

**Supplementary Figure S7:** *In Silico* analysis of Hsc70 promoter Cis regulatory elements available in the PLACE database (<https://www.dna.affrc.go.jp/PLACE>)

**Supplementary Figure S8:** Clustal alignment of deduced amino acid sequences of all *Hsc70* alleles in 13 wild potato accessions.

**Supplementary Table 1:** Tuber yield GWAS significant marker-trait associations (MTAs) from all genetic effect and GWAS models employed in the study.

**Supplementary Table 2:** List of wild potato accessions used in this study.
